# A Machine Learning Framework Identifies Plastid-Encoded Proteins Harboring C_3_ and C_4_ Distinguishing Sequence Information

**DOI:** 10.1093/gbe/evad129

**Published:** 2023-07-18

**Authors:** Nilanth Yogadasan, Andrew C Doxey, Simon D X Chuong

**Affiliations:** Department of Biology, University of Waterloo, Waterloo, ON, Canada; Department of Biology, University of Waterloo, Waterloo, ON, Canada; Department of Biology, University of Waterloo, Waterloo, ON, Canada

**Keywords:** C_4_ photosynthesis, convergent evolution, PACMAD, grasses, plastome, machine learning

## Abstract

C_4_ photosynthesis is known to have at least 61 independent origins across plant lineages making it one of the most notable examples of convergent evolution. Of the >60 independent origins, a predicted 22–24 origins, encompassing greater than 50% of all known C_4_ species, exist within the Panicoideae, Arundinoideae, Chloridoideae, Micrairoideae, Aristidoideae, and Danthonioideae (PACMAD) clade of the Poaceae family. This clade is therefore primed with species ideal for the study of genomic changes associated with the acquisition of the C_4_ photosynthetic trait. In this study, we take advantage of the growing availability of sequenced plastid genomes and employ a machine learning (ML) approach to screen for plastid genes harboring C_3_ and C_4_ distinguishing information in PACMAD species. We demonstrate that certain plastid-encoded protein sequences possess distinguishing and informative sequence information that allows them to train accurate ML C_3_/C_4_ classification models. Our RbcL-trained model, for example, informs a C_3_/C_4_ classifier with greater than 99% accuracy. Accurate prediction of photosynthetic type from individual sequences suggests biologically relevant, and potentially differing roles of these sequence products in C_3_ versus C_4_ metabolism. With this ML framework, we have identified several key sequences and sites that are most predictive of C_3_/C_4_ status, including RbcL, subunits of the NAD(P)H dehydrogenase complex, and specific residues within, further highlighting their potential significance in the evolution and/or maintenance of C_4_ photosynthetic machinery. This general approach can be applied to uncover intricate associations between other similar genotype-phenotype relationships.

**Significance**

The molecular determinants of C_4_ evolution, although extensively studied, remain largely unclear. A collective interest in these determinants is driven by their potential to guide efforts of engineering the formidable C_4_ photosynthetic pathway into key crop species. Several studies have uncovered associations between specific genomic and proteomic changes with C_3_–C_4_ transitions, but few have focused on plastomic associations. By uniquely employing a machine learning framework to screen plastomic information for C_3_ and C_4_ distinguishing features we have identified key sites and sequences undergoing changes associated with the C_3_–C_4_ transition in grasses. Presently identified sites include those yet-to-be-discussed meaningfully in the field, and we begin to outline here their potential significance in driving C_4_ evolution.

## Introduction

The C_4_ photosynthetic pathway arose as an adaptation to falling atmospheric carbon dioxide (CO_2_) levels approximately 30 million years ago. Changes in the anatomy and metabolism of C_4_ plants allowed for CO_2_ concentration to take place in photosynthetic leaf tissue and cells, thereby inhibiting the negative effects of CO_2_ limitation (Sage et al. 2012). Under high temperature, high light and/or dry conditions, limited CO_2_ availability is further exacerbated by competition with oxygen (O_2_) for fixation by the key photosynthetic enzyme Rubisco and this is suggested to have led to the evolution, expansion, and diversification of C_4_ plant species across warmer climate regions (Bouchenak-Khelladi et al. 2009; Watcharamongkol et al. 2018). With only ∼3% (∼8,500 species) of all plant species employing C_4_ metabolism, C_4_ plant species are responsible for ∼23% of terrestrial gross primary production and this is in large part due to their outperformance of the ancestral C_3_ state in uncovered, hot to warm and arid regions (Still et al. 2003; Sage et al. 2012; Atkinson et al. 2016).

Despite extensive knowledge of the existence of various C_4_ pathways and a general understanding of how these pathways achieve and utilize CO_2_ concentration to negate photorespiration, exact genomic determinants and enablers of C_4_ evolution remain to be uncovered (Christin et al. 2007; Hibberd and Covshoff 2010; Gowik et al. 2011; Bianconi et al. 2020). Due to the complexity of the C_4_ trait, it has been suggested that changes to, potentially, hundreds of genes would be necessary for its evolution (Hibberd and Covshoff 2010; Sage 2017). Coordinated changes between the plastid and nuclear genomes (plastid genome hereafter referred to as plastome) would also be required to establish the necessary regulatory mechanisms governing the differences in plastid gene expression between C_3_ and C_4_ photosynthesis (Woodson and Chory 2008; Hibberd and Covshoff 2010). With all this in mind, it is surprising to note that C_4_ photosynthesis has evolved independently at least 61 times, making the trait a leading example of convergent evolution (Sage 2017).

Several studies have previously investigated evolutionary dynamics and selection acting on individual protein-encoding genes with known altered functionality and/or regulation during C_4_ photosynthesis compared to C_3_ (Hibberd and Covshoff 2010). These include genes encoding Rubisco small subunit (*rbcS*) (Berry et al. 2016), Rubisco large subunit (*rbcL*) (Kapralov and Filatov 2007; Christin et al. 2008; Kapralov et al. 2012, Piot et al. 2018), phosphoenolpyruvate carboxylase (Christin et al. 2007; Paulus et al. 2013; Rosnow et al. 2014; Moody et al. 2020), carbonic anhydrases (Tanz et al. 2009), C_4_-acid decarboxylases and more (Huang et al. 2017, Bianconi et al. 2020). However, apart from the plastome-encoded *rbcL* gene, little work has gone into uncovering selection and adaptation events associated with C_4_ photosynthesis and the C_3_—C_4_ photosynthetic transition within plastomes (Christin et al. 2008; Piot et al. 2018; Casola and Li 2022). The focus on *rbcL* has led to the identification of sites under strong positive selection in this gene that is, at least in part, driven by a C_3_—C_4_ photosynthetic shift (Christin et al. 2008; Kapralov et al. 2011; Kapralov et al. 2012; Piot et al. 2018; Casola and Li 2022). The existence of this positive selective pressure on one plastome-encoded gene suggests the potential for similar pressures being exerted at other sites in the plastome. Identified substitutions associated with the metabolic shift could be functionally and evolutionarily relevant in the establishment and maintenance of C_4_ photosynthesis.

In addition to the globe's most economically important crops—including rice, wheat, and corn—the grass family (Poaceae) provides a unique set of species from which a study of C_3_–C_4_ transitions can be conducted. Greater than 50% of all known C_4_ species belong to the Panicoideae, Arundinoideae, Chloridoideae, Micrairoideae, Aristidoideae, and Danthonioideae (PACMAD), clade found within the Poaceae family (Edwards et al. 2010; Christin et al. 2013). The sister Bambusoideae, Ehrhartoideae, Pooideae clade contains roughly the same number of species all of which utilize C_3_ photosynthesis (Edwards et al. 2010; GPWG II 2012). Of the, at minimum, 61 origins of the C_4_ trait, a predicted 22–24 origins exist within the PACMAD clade alone (GPWG II 2012). In this study, we assess and compare the abilities of plastomic protein-coding sequences in providing C_3_ and C_4_ distinguishing information to a machine learning (ML) model for subsequent classification. With several C_4_ origins in grasses, we were able to compile a dataset of plastomes from C_4_ and closely related C_3_ species to ensure patterns identified by our models are due to photosynthetic type instead of lineage. After screening for genes that inform highly accurate classifiers, we determine the specific sites within the region sequence data that are most strongly influencing our classification models. The identified C_3_/C_4_ determining combinations of sites are potential changes—parallel, convergent, or otherwise—associating with photosynthetic type.

While there are several biochemical, anatomical, and physiological methods to determine the C_3_/C_4_ status of a plant species, our ML pipeline employed here does not aim to classify species but rather to answer 1) whether a photosynthetic type can be predicted from specific sequence information alone; and 2) what sequence features (genes, residues) enable the accurate prediction of C_3_/C_4_ phenotype and are, therefore, likely subject to key driving and/or adaptive forces separating C_3_ and C_4_ metabolism. Our framework answers these questions and, by the very nature of ML, uniquely arrives at patterns of changes associated with photosynthetic type by interpreting entire protein, and even whole complex, sequence data. This is in contrast to conventionally applied selection tests (Christin et al. 2008; Piot et al. 2018; Casola and Li 2022) which examine parallel/convergent associations on a residue-by-residue basis and independently of one another. By analyzing patterns across a functional unit (complex, subunit, protein, domain, motif, etc.), biologically relevant phenomena such as complementary mutations, compensatory mutations, functional redundancies, and more can be accounted for. However, our ML framework cannot provide insight into the selective pressures acting on identified associations and is not meant as a replacement for conventional selection testing. Instead, our work here offers alternative insight into plastomic associations with photosynthetic type from the unique perspective granted by the nature of ML and classification modeling. On a more expansive PACMAD dataset, than ones previously considered (Piot et al. 2018; Casola and Li 2022), we employed our ML framework and identified plastidic complexes, proteins, and patterns of substitutions within, capable of informing highly accurate C_3_/C_4_ classifiers. These include both known, via positive selection testing, and yet-to-be-considered sites and genes worth investigating further for a greater understanding of mechanisms driving the evolution of C_4_ photosynthesis.

## Results

### Plastomic Screen for Protein Sequences Informing Accurate C_3_/C_4_ Classification Models

**Fig. 1.**
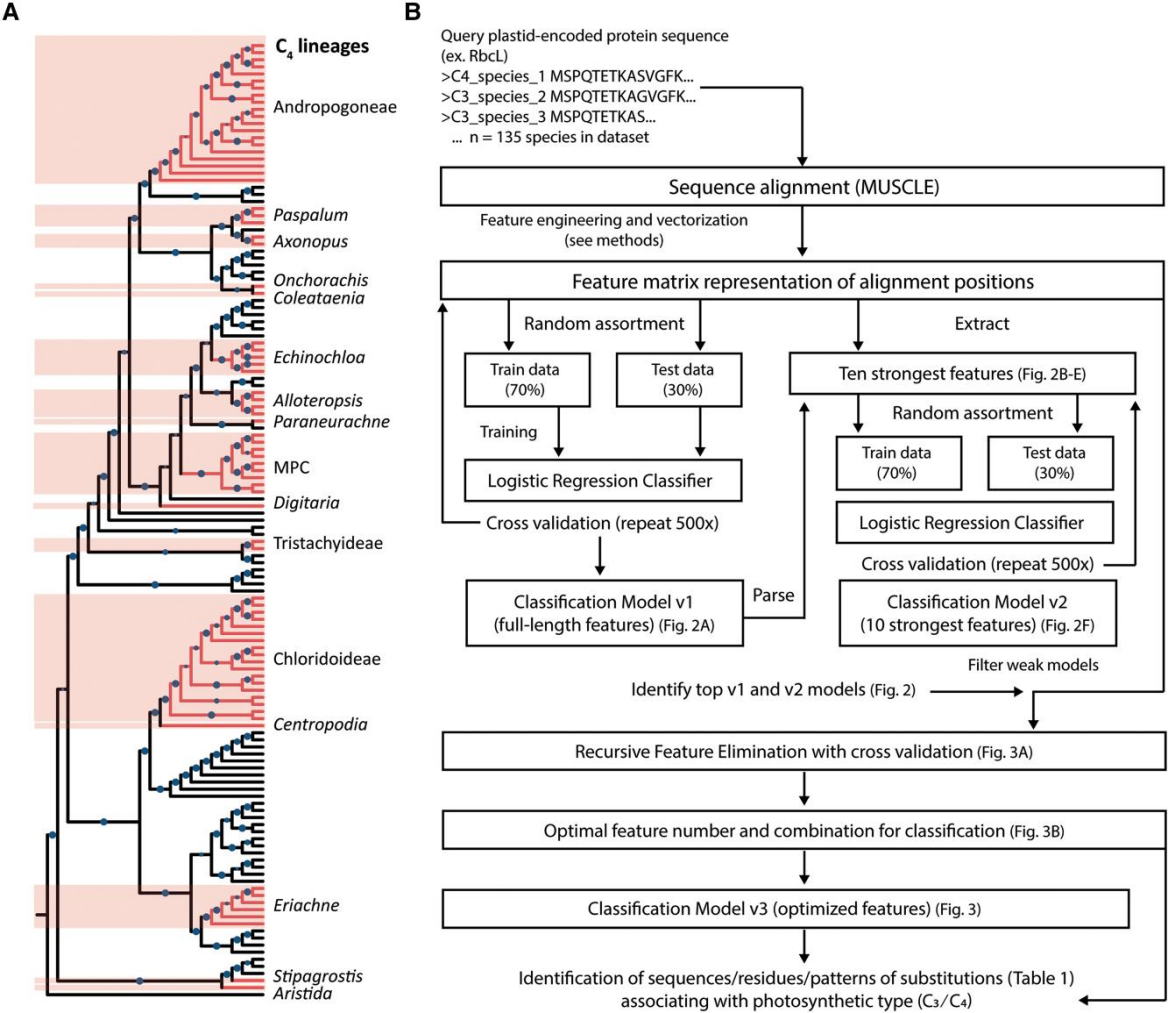
Overview of ML framework employed to screen PACMAD plastomes for C_3_ and C_4_ distinguishing information. (*A*) Cladogram of 134 C_3_ and C_4_ PACMAD species from our assembled dataset (supplementary table S1, Supplementary Material online). Phylogeny was obtained using RAxML (GTR+Γ model) (Stamatakis 2014) from concatenated alignments of plastid marker genes (*rbcL*, *ndhF*, *matK, rpoC2, psaA, psaB, psaI, rpl32*, and *ccsA*) and rooted to *Anomochloa marantoidea*, a grass species from outside of the PACMAD clade. Independent C_4_ lineages, as previously reported, are labeled. Shaded and unshaded branches indicate C_4_ lineages and C_3_ lineages, respectively. The size of circles represents bootstrap support. (*B*) Pipeline employed for identification of residues and substitution patterns in plastid protein sequences from the PACMAD clade associating with photosynthetic type.

With our assembled dataset of 134 PACMAD species, consisting of 76 C_4_ species and 58 C_3_ species (supplementary table S1, Supplementary Material online), we screened each of 76 plastid-encoded protein sequences based on their ability to train an accurate C_3_/C_4_ ML logistic regression classification model. Our dataset encompasses 16 different C_4_ lineages (fig. 1*A*, supplementary table S1, Supplementary Material online) with independent C_4_ origins (GPWG II 2012). A phylogenetic tree of the species in our dataset (fig. 1*A*) highlights the independent origins as well as the intent of the dataset curation; to include plastomes from C_4_ and closely related C_3_ species to ensure pattern identification and classification are driven by independent C_3_/C_4_ transitions instead of phylogenetic bias due to common ancestry. This tree is largely congruent with previous studies of the PACMAD clade (GPWG II 2012; Casola and Li 2022; Huang et al. 2022). Figure 1*B* illustrates the general framework employed in this study to first arrive at an unbiased summary of plastid-encoded protein sequences capable of informing accurate C_3_/C_4_ classification models, followed by algorithmic methods used to identify specific residues within each sequence that are most heavily influencing classification decisions.

**Fig. 2.**
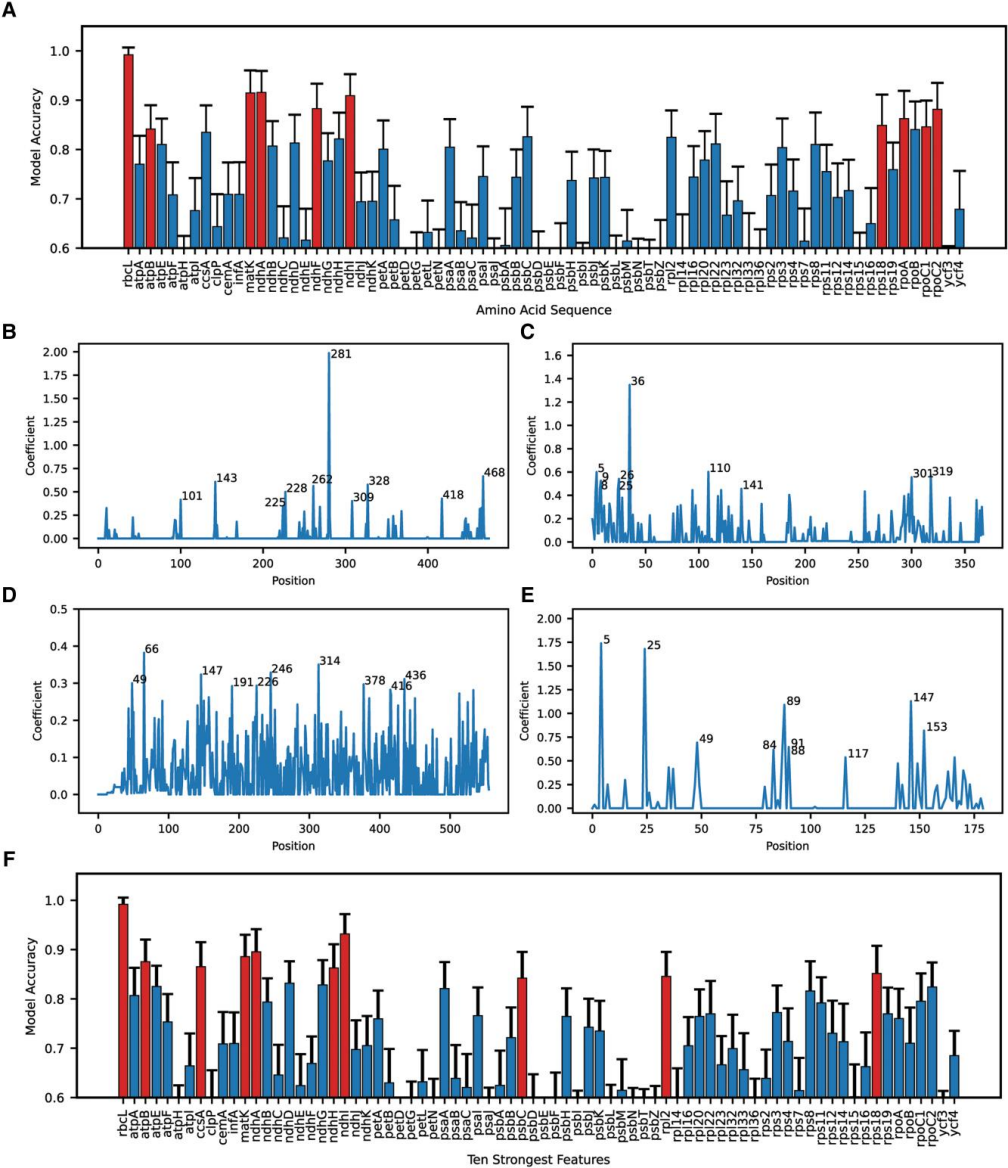
Performance of C_3_/C_4_ classification models trained on plastid-encoded, protein sequence data from PACMAD grasses. (*A*) Average C_3_/C_4_ classification accuracy, after cross validation (with repeated random subsampling, *n* = 500), of models trained on each of 76 plastid-encoded, full-length protein sequences (70:30 train:test split) from 134 Poaceae species. Red bars highlight the ten best-performing classifiers. Absolute values of the regression coefficients associated with (*B*) RbcL alignment positions, (*C*) NdhA alignment positions, (*D*) MatK alignment positions, and (*E*) NdhI alignment positions from the initial (*A*) classification models are plotted as examples of the initial feature selection method. The ten highest peaks corresponding to the ten strongest features from each model are labeled. (*F*) Average C_3_/C_4_ classification accuracy, after cross validation (*n* = 500), of models trained on identified ten strongest features from each of 76 plastid-encoded, protein sequences from 134 Poaceae species (70:30 train:test split). Red bars highlight the ten best-performing classifiers.

Full-length protein sequences from each species of our dataset were aligned and converted to feature sets for training, testing, and validation of a classification model (classification model v1 in fig. 1*B*, see methods for details). Of the 76 plastid-encoded proteins screened via classification model v1, the best predictor of C_3_/C_4_ status is RbcL (fig. 2*A*). Using RbcL amino acid (AA) sequence data, our v1 classification model can correctly classify species as C_3_ or C_4_ with >99% accuracy, on average, after cross-validation. The top ten performing v1 models are based on the full-length sequences from RbcL, NdhA, MatK, NdhI, NdhF, RpoC2, RpoA, Rps18, RpoC1, and AtpB. Each of these models correctly classifies C_3_ and C_4_ species of our dataset of 134 PACMAD species with greater than 84% accuracy (fig. 2*A*). These models perform significantly better (*P* < 0.001) than the performance achieved by 80% of v1 models. Of note, apart from RbcL, are the performances of v1 models built on NdhA, MatK, and NdhI which each score classification accuracies greater than 90% and perform significantly better (*P* < 0.001) than the next best-performing model built on NdhF.

The 10 strongest features, corresponding to 10 alignment positions most heavily influencing classification in each v1 model, were identified (fig. 2*B*-*E* four examples shown, supplementary table S2, Supplementary Material online) and then used exclusively to train our v2 classification models (classification model v2 in fig. 1*B*). Our v2 models, making use of only ten features per model, are not subject to the same potential for overfitting as our v1 models where the number of features is equivalent to the number of positions in each sequence alignment. Restricting feature size to below the number of independent C_4_ lineages in our dataset also limits the ability of our models to rely on phylogenetic signals for classification.

The ten strongest features from RbcL, NdhI, NdhA, MatK, AtpB, CcsA, NdhH, Rps18, Rpl2, PsbC all train v2 models with classification accuracies >84% and perform significantly better (*P* < 0.001) than 80% of all v2 models (fig. 2*F*). The ten strongest features alone from RbcL are enough to inform a classifier with >99% accuracy making it the strongest performing v2 model as well. Again, of note is the v2 model built on the ten strongest features from NdhI which has a classification accuracy of 93.2%, after cross validation. The identification of v2 models that perform classification with >90% accuracy from a restrictive feature set, that is an order of magnitude smaller (<10%) than our sample size and is also fewer than the number of independent C_4_ lineages in our dataset, strongly suggests informative ability that is likely linked to biology and is not a product of training biases or overfitting. This being said, screening with v2 models would be the most stringent, of our study, in identifying potentially informative sequences and sequence information. As such, only sequence information from RbcL and NdhI informed v2 models with >90% accuracy.

### Recursive Feature Elimination to Identify Optimal Residue Combinations Informing Accurate Classification Models

**Fig. 3.**
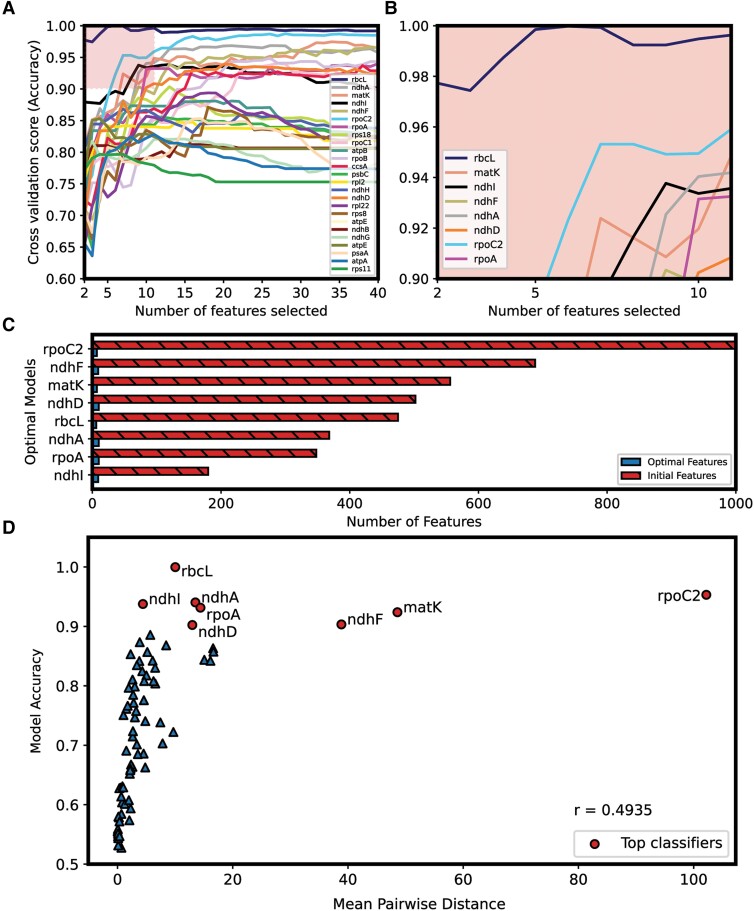
RFE-CV to identify key feature combinations informing top C_3_/C_4_ classification models. **(***A***)** Cross validation (repeated random subsampling, *n* = 500) scores of classification models informed, initially, by full-length alignments and recursively modeled with a single identified least valuable feature (alignment position) removed in each iteration until a minimum of two features remained. The boxed and highlighted region corresponds to (*B*) which encompasses an “optimal” zone where models are built on ten or fewer features and have classification accuracy scores greater than or equal to 0.9 or 90%. (*C*) Comparison of a number of initial features used for classification to a number of features after optimal feature selection. (*D*) The average accuracy of the best-performing models after RFE-CV, from iterations making use of <11 features, is plotted against MPD from corresponding sequence alignments, and Pearson's correlation coefficient (*r*) is determined.

Our v1 models (fig. 2*A*) were trained on entire protein sequence alignment data where each position in the alignment acted as a feature to be used for classification modeling. With an RbcL alignment of length 475, our RbcL v1 model would have, therefore, been trained on 475 features. With a dataset of 134 PACMAD species there exists a potential for overfitting due to a number of features being much greater than the dataset size (Vabalas et al. 2019; Chen et al. 2020). Our v2 models (fig. 2*F*) were trained on the ten strongest features, or ten alignment positions most heavily influencing classification, parsed from one iteration of each v1 model. By employing recursive feature elimination with cross validation (RFE-CV), a robust algorithm by which classification models are recursively built using fewer features in each iteration by dropping the previous iteration's weakest feature (Guyon et al. 2002; Arif et al. 2020), we were able to identify optimal feature combinations from each of the sequences informing top-performing v1 and v2 models (fig. 3).

RbcL, MatK, NdhI, NdhF, NdhA, NdhD, RpoA, and RpoC2 are the only sequences identified to possess a subset of ten or fewer features that can inform a classifier with >90% average accuracy after cross validation. The minimal feature usage (fig. 3*C*) and high classification accuracies of our optimal v3 models (classification model v3 in fig. 1*B*) more confidently, but with less stringency than v2 models, suggest an association between the corresponding protein sequences and C_3_/C_4_ status.

To assess the propensity of our optimized feature models to rely on initial sequence alignment length or variation across species, we determined correlations of these factors with model accuracy. Accuracy of the top-performing model—identified from iterations making use of <11 features after RFE-CV—for each sequence was plotted against respective mean pairwise distance (MPD) (fig. 3*D*) calculated from corresponding alignments. C_3_ sequences were used exclusively to determine MPD so that variation due to C_3_–C_4_ photosynthetic transitions, which is expected to correlate to top model performance, was not captured. MPD is used here as a measure of the amount of information inherently present in queried alignments due to length and variation. Low-moderate correlation (*r* = 0.4935) of model accuracy with MPD was noted. Individually, both length (supplementary fig. S1*A*, Supplementary Material online) (*r* = 0.56) and variation (supplementary fig. S1*B*, Supplementary Material online) (*r* = 0.55) exhibited moderate levels of positive correlation with optimal model accuracy. However, neither factor exclusively dictated classification ability. Our optimized RbcL model was our strongest performing model despite ten other models trained on lengthier sequence alignments and 31 other models with greater variation/noise across species for coincident pattern detection. NdhI informed our third best-performing model despite 32 lengthier sequence alignments and 27 alignments with greater variation across sequences to learn from. The top performance of these models despite being provided with comparatively less information for pattern detection suggests an identification of C_3_/C_4_ specific signals and a lack of reliance on phylogenetics-associated sequence information. The high accuracy scores of optimal RpoC2, MatK, and NdhF models, however, are potentially artifacts of the corresponding sequence alignments being among the lengthiest and most variable (fig. 3*D*, supplementary fig. S1, Supplementary Material online) of all sequences assessed.

A subset of only six features from RbcL, identified via RFE-CV, is capable of informing a 99.98% accurate C_3_/C_4_ classifier, after cross validation. Specific residues at 101, 143, 281, 309, 418, and 468 (numbered according to positions in *Zea mays* RbcL) are all that is necessary for near-perfect classification, according to all relevant classification metrics, of PACMAD species in our dataset by photosynthetic type (table 1). Nine and ten feature subsets were identified as the optimal combinations from NdhI and NdhA, respectively, with both optimal models achieving >94% accuracy. Ten features from RpoA and ten features from NdhD were used to inform corresponding optimal models, both of which achieve accuracy scores >90% (table 1). Again, quite interestingly, three out of five of the top v3 models (not including potentially inflated performances from RpoC2, MatK, and NdhF) are trained on optimized features from sequences encoding subunits of the NDH complex. This enrichment of NDH subunits being identified among top informers of our v1, v2, and v3 classification models strongly implicates the NDH complex in roles related to, and likely differing between C_3_ and C_4_ metabolism.

**Table 1:** Summary of Top Performing v3 Models After Feature Selection Using RFE-CV

AA seq	Optimal Features (ap)	Residue Positions (*Zm*)	ROC AUC	Precision	Recall	F1	Acc
RbcL	101, 143, 281, 309, 418, 468	101, 143, 281, 309, 418, 468	1.0	1.0	0.9997	0.9998	0.9998
RpoC2	357, 368, 450, 774, 1050, 1132, 1290	357, 368, 450, 706, 942, 1011, 1151	0.9639	0.9728	0.9471	0.9591	0.955
NdhI	5, 25, 38, 49, 84, 88, 89, 147, 153	5, 25, 38, 49, 84, 88, 89, 147, 153	0.9583	0.9784	0.9197	0.9464	0.9423
NdhA	5, 29, 36, 98, 110, 187, 298, 301, 319, 320	5, 27, 34, 96, 108, 184, 293, 296, 314, 315	0.9805	0.9910	0.9031	0.9436	0.9401
RpoA	6, 14, 146, 163, 180, 243, 279, 326, 329, 336	6, 14, 146, 161, 176, 237, 270, 317, 327	0.969	0.9444	0.9305	0.9359	0.9295
MatK	49, 147, 159, 314, 378, 417, 436	16, 111, 123, 274, 338, 377, 396	0.9673	0.8491	0.9716	0.93	0.9191
NdhD	64, 76, 114, 334, 364, 376, 442, 451, 497, 501	62, 74, 112, 332, 362, 374, 440, 449, 495, 499	0.963	0.9214	0.9197	0.9183	0.9089
NdhF	89, 145, 287, 340, 400, 566, 568, 597, 659	89, 145, 287, 340, 400, 555, 557, 586, 646	0.9653	0.9860	0.8497	0.9114	0.9082

ap—positions numbered according to alignment position.

*Z. m—*Residue positions numbered according to positions in *Zea mays* protein sequence.

Average scores of classification metrics (ROC AUC, Precision, Recall, F1, Accuracy) after cross validation (using repeated random subsampling, *n* = 500, 70/30 train/test split) are shown. Gray cells indicate models and model performances that are potential artifacts of corresponding sequence length/variation. ROC AUC, area under receiver operating characteristic curve.

Average accuracy and AUROC (area under receiver operating characteristic curve) scores were determined after cross-validation using repeated random subsampling (*n* = 500) for each v1, v2, and v3 model. These were compared to scores achieved by models trained using the same sequence information (feature data) with randomized C_3_ and C_4_ labels. Supplementary figure S2, Supplementary Material online summarizes the evaluation of our top optimal v3 models. The top-performing models, all have significantly higher accuracy and AUROC scores (*P* < 0.001, Student's *t*-test) than the associated model trained on randomized labels (supplementary fig. S2, Supplementary Material online). This is further confirmation that our top model performance is not a product of overfitting and that our ML framework is likely picking up on biologically relevant patterns in the sequence data rather than relying on coincident patterns expected to be found in datasets with many input features.

### Combined Subunit Classification Modelling to Assess Entire Plastidic Complexes

**Fig. 4.**
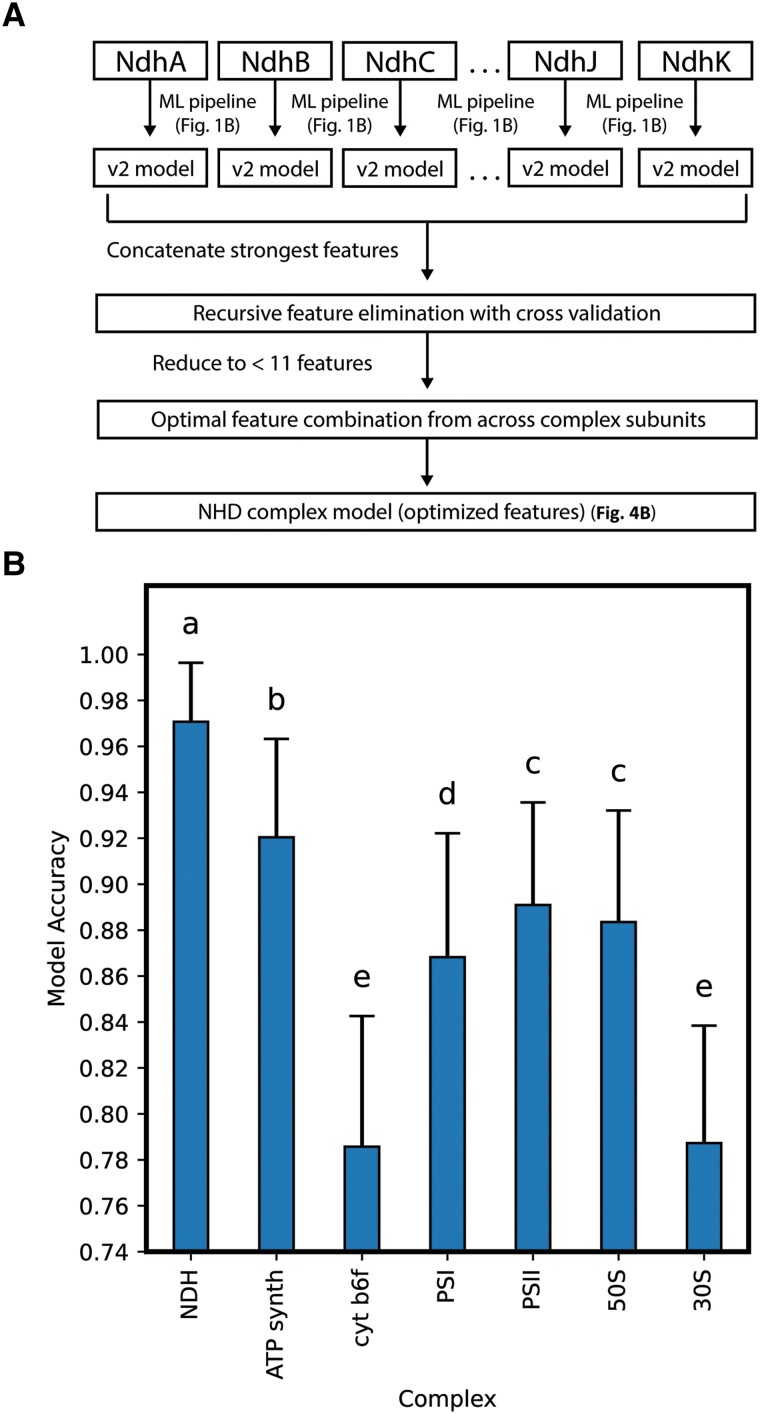
Performance of C_3_/C_4_ classification models trained on optimal features selected from plastidic, multimeric complexes. (*A*) Pipeline employed for generating optimal feature combinations from across all plastid-encoded subunits making up a specific plastidic complex. NDH complex is used here as an example. (*B*) Average C_3_/C_4_ classification accuracy of the top-performing model with ten or fewer optimal features after RFE-CV from each of seven plastidic complexes. NDH—NAD(*P*)H Dehydrogenase complex, ATP Synth—ATP synthase, cyt b6*f*—cytochrome b6*f* complex, PSI—Photosystem I, PSII—Photosystem II, 50*S*—50*S* ribosomal subunit, 30*S*—30*S* ribosomal subunit. Significance was determined using Student's *t*-tests (*P* < 0.001) with Bonferroni correction and significant differences are denoted by letters.

To assess whether entire plastidic complexes harbor C_3_/C_4_ distinguishing information, we expanded upon our ML framework to determine whether accurate classification models could be trained on optimal features from across constituent plastid-encoded subunits (fig. 4). We assessed seven plastidic complexes, each of which including at least five different plastid-encoded subunits, for their abilities to inform accurate classifiers. The plastid *ndh* genes (*ndhA—ndhK*) encode 11 different subunits that are part of the NAD(P)H dehydrogenase complex (hereafter NDH complex) (Ifuku et al. 2011; Laughlin et al. 2020), the plastid *atp* genes (*atpA, atpB, atpE, atpF, atpH, atpI*) encode 6 different subunits of the ATP-synthase complex (Hennig and Herrmann 1986), the plastid *pet* genes (*petA, petB, petD, petG, petL, petN*) encode six different subunits of the cytochrome b_6_f complex (Stroebel et al. 2003), the plastid *psa* genes (*psaA, psaB, psaC, psaB, psaJ*) encode five different subunits included in the Photosystem I (PSI) supercomplex (Nelson and Yocum 2006), the plastid *psb* genes (*psbA—psbF, psbH—psbM, psbT, psbZ*) encode 14 different subunits included in the Photosystem II (PSII) supercomplex (Nelson and Yocum 2006), and the plastid *rps* and *rpl* genes typically encode 12 proteins for the small ribosomal subunits, and 9 proteins for the large ribosomal subunits respectively (Yamaguchi and Subramanian 2000; Yamaguchi and Subramanian 2003). By employing the pipeline summarized in figure 4*A* we were able to build classification models for each of the plastidic complexes based on optimal features selected from across their respective plastid-encoded subunits. In short, we obtained the strongest feature subset (v2 model features) for each of the subunits (as outlined in fig. 1*B*), concatenated these feature sets according to complex, and used RFE-CV to arrive at an optimal subset of ten or fewer features per complex for C_3_/C_4_ classification.

Figure 4 *B* compares the top-performing models, of ten or fewer features, for each plastidic complex. In agreement with what has been hinted at by our individual sequence models, NDH complex sequence information is capable of informing highly accurate classification models. With an average accuracy of 97.07% after cross validation, the NDH complex model performs significantly better (*P* < 0.001, Student's *t*-test) than all six other models trained on optimized, minimal (ten or fewer) feature sets from queried complexes. No other complex achieved average accuracies >92% (table 2). This significant difference in the informative power of the NDH complex versus other major plastid complexes suggests a specific association with factors driving C_4_ evolution. The NDH complex model also performs significantly better (*P* < 0.001, Student's *t*-test) than any model trained on data from its individual constituent subunits, while still making use of a minimal feature set.

**Table 2:** Summary of Optimized Plastidic Complex Models and Identified Key Residues

Complex	Combined Subunits	Optimal Features (Subunit-ap)	Optimal Residues (Subunit-*Zm*)	Acc
NDH (*ndh*)	A,B,C,D,E,F,G,H,I,J,K	A-5, A-36, A-319, D-114, H-274, I-25	A-5, A-34, A-314, D-112, H-269, I-25	0.9707
ATP synthase (*atp*)	A,B,E,F,H,I	B-96, B-105, B-383, B-450, E-55, E-102, E-106, E-130, F-14, I-92	B-96, B-105, B-383, B-450, E-55, E-102, E-106, E-130, F-14, I-92	0.9190
Cyt b_6_f (*pet*)	A,B,D,G,L,N	A-5, A-20, A-26, A-63, A-145, A-177, D-17, L-19, L-29, N-5	A-5, A-20, A-26, A-63, A-145, A-177, D-3, L-19, L-29, N-3	0.7857
PSI (*psa*)	A,B,C,I,J	A-34, A-310, A-484, B-492, C-29, I-4, I-5, I-24	A-34, A-310, A-484, B-492, C-29, I-24, I-5, I-24	0.8682
PSII (*psb*)	A,B,C,D,E,F,H,I,J,K,L,M,T,Z	B-182, C-191, C-221, C-291, C-441, D-5, E-79, K-8, K-41, K-47	B-182, C-177, C-207, C-277, C-427, D-5, E-79, K-8, K-39, K-45	0.8909
50S (*rpl*)	2,14,16,20,22,23,33,36	2–185, 14–67, 16–120, 16–126, 16–142, 20–109, 22–103, 22–114, 23–92, 33–4	2–179, 14–67, 16–113, 16–119, 16–135, 20–103, 22–102, 22–113, 23–92, 33–4	0.8835
30S (*rps*)	2,3,4,7,8,11,12,14,15,16,18,19	3–118, 3–174, 3–179, 3–197, 11–13, 11–122, 14–14, 16–55, 16–37, 16–48	3–105, 3–161, 3–166, 3–184, 11–13, 11–122, 14–14, 16–54, 16–36, 16–47	0.7873

Subunit-ap—positions numbered according to alignment positions from the specific subunit.

Subunit-*Zm*—positions numbered according to protein sequence positions of the specific subunit in *Zea mays*.

Acc-average accuracy after cross validation (using repeated random subsampling, *n* = 500, 70/30 train/test split).

NDH, NAD(P)H Dehydrogenase complex; Cyt b6f, cytochrome b6f complex; PSI, Photosystem I; PSII, Photosystem II; 50S, 50S ribosomal subunit; 30S, 30S ribosomal subunit.

### Assessment of Key Residue Combinations Informing C_3_/C_4_ Classification

Of all models (v1, v2, v3, and complex) trained, tested, and validated in our study, the models informed by optimal features from RbcL and the NDH complex are the strongest performing in terms of accuracy and all relevant classification metrics. Six features, or sites, from RbcL (table 1) and six combined features from plastid-encoded subunits of the NDH complex (table 2) inform classifiers with 99.98% and 97.07% accuracy, respectively. These both perform significantly better (*P* < 0.001, Student's *t*-test) than the next best model, of all considered, which is informed by optimal features from the individual NdhI subunit (94.23% average accuracy). Supplementary figure S3, Supplementary Material online maps all of the residues at each optimal feature position (relative to *Zea mays* orthologous sequences) from these top two performing models to the corresponding species of our PACMAD dataset.

Position 281 from RbcL is associated with the most consistent parallel substitution that had taken place during C_3_–C_4_ photosynthetic transitions of all features identified in our study. In all C_3_ species considered the residue aligned with position 281 is an alanine (A), while for >66% of C_4_ species in our dataset, the corresponding residue is a serine (S) and this A-281-S substitution is observed in 15 of the 16 C_4_ lineages examined. Similarly parallel are the changes at identified optimal feature positions 101, 143, 309, and 468 of RbcL. Residues aligned with position 101 are valine (V) for >98% of C_3_ species, but <56% of C_4_ species. Isoleucine (I) appears at this position in ∼44% of C_4_ species, and in 11 of 16 C_4_ lineages, but in only a single C_3_ species. All C_3_ species of our dataset have threonine (T) for residues aligned with position 143, but >64% of C_4_ species from across five C_4_ lineages have alanine (A) instead. Although methionine (M) appears at position 309 in the majority of C_3_ (100%) and C_4_ (∼65%) species of our dataset, an M-309-I substitution is only found in C_4_ species and across 11 different lineages. Residues aligned with position 468 are glutamic acid (E) for >96% of C_3_ species but only ∼30% of C_4_ species. Aspartic acid (D) appears instead in ∼70% of C_4_ species, across eight C_4_ lineages, and yet in only a single C_3_ species. Position 328, while not included as one of the optimal six features in our RbcL v3 model, had been one of the ten strongest features identified from our RbcL v1 model (fig. 2*B*) and exhibits notable parallel changes associated with C_3_—C_4_ transition >86% of C_3_ species of our dataset have alanine (A) aligned with position 328, while serine (S) appears instead in >78% of C_4_ species and in 12 of 16 C_4_ lineages.

Of the six optimal features informing our NDH complex model, the most notable level of parallel changes associated with the C_3_—C_4_ transition was observed at position 25 of the NdhI subunit. Here, serine (S) is present in >86% of C_3_ species and <16% of C_4_ species. Glycine (G) is the predominant C_4_ residue at this position, as it is present in >84% of C_4_ species, across 12 C_4_ lineages. Less notable, but relevant, changes associated with photosynthetic type are found at identified optimal feature positions 36 of the NdhA subunit and 274 of the NdhH subunit. ∼95% of C_3_ species and ∼65% of C_4_ species have threonine (T) as the residue corresponding to NdhA-36, but alanine (A) is found instead in ∼35% of C_4_ species, appearing in five lineages, and only 2 C_3_ species. The residues corresponding to NdhH-274 in ∼95% of C_3_ species and only ∼60% of C_4_ species is arginine (R). Arginine (R) is substituted with either lysine (K), glycine (G), or glutamic acid (E) in ∼40% of C_4_ species, across five lineages, while the only change from arginine across our C_3_ dataset is to lysine in three species.

**Fig. 5.**
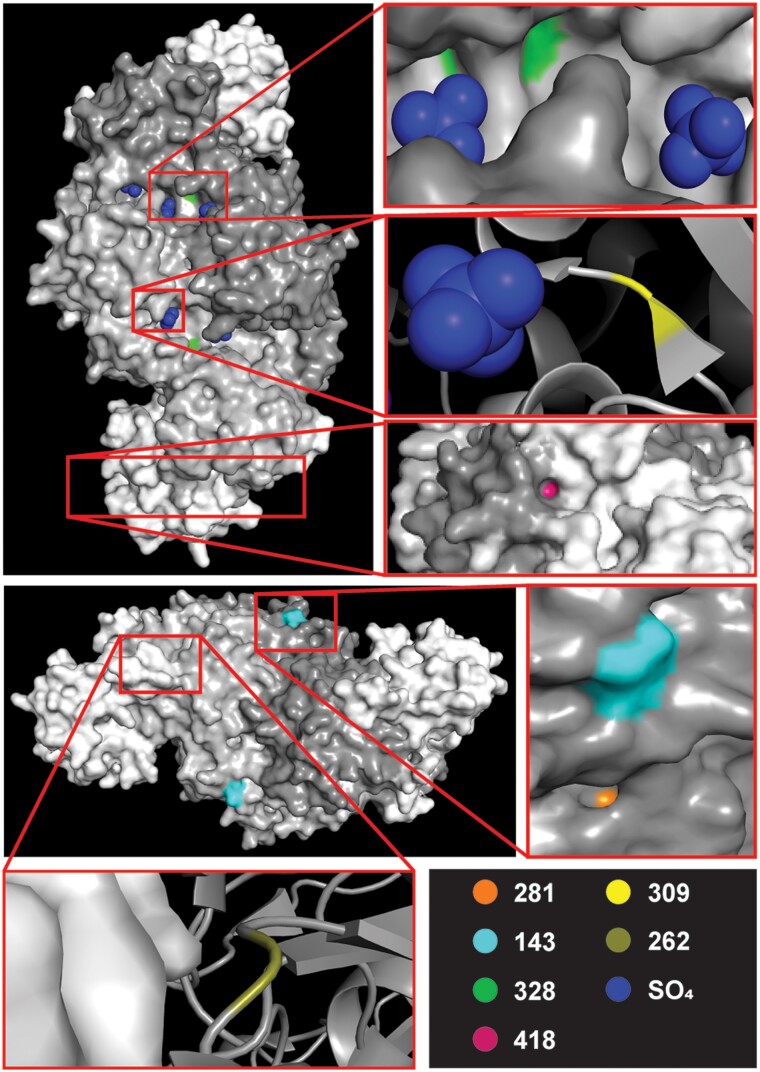
Structure of rice (*Oryza sativa*) Rubisco in complex with sulfate ions (Matsumura, et al. 2020) (PDB id: 6KYI). The strongest and optimal feature residues encoded by *rbcL* that are surface exposed, at subunit interfaces and/or near the active site are highlighted. Graphics were obtained using PyMol (DeLano 2002).

Based on the crystal structure of rice (*Oryza sativa)* Rubisco (Matsumura et al. 2020) (Protein Data Bank [PDB] id: 6KYI), six of the ten strongest RbcL features (v2 model)—including four of the six identified optimal (v3 model) features—residues 143, 262, 281, 309, 328, and 418 of RbcL are surface exposed, interface proximal and/or active site proximal amino acids (fig. 5). Residue 262, although not exposed as part of the Rubisco complex, resides on the exterior of Rubisco LSU and is found at the interface of the LSU and Rubisco SSU. Residue 418 also lies at the interface between the LSU and SSU. Residue 328 is exposed and neighbors a sulfate-bound site which represents an active site of RbcL where the phosphate group of RuBP would normally bind (Matsumura et al. 2020). Residue 309 resides on the interior of RbcL and next to a RuBP binding site.

## Discussion

The objective of our study was to compare the abilities of specific plastid-encoded protein sequence information to inform accurate ML C_3_/C_4_ classification models. We converted sequence alignments to feature sets for modeling and then, as part of a progressively optimizing pipeline, assessed full-length sequences (v1 models), selected features (v2 models) and algorithmically obtained “optimal” feature sets (v3 models) for classification ability. RbcL came out as the top informer at every iteration of modeling in our framework. Sequence data from RbcL alone was all that was necessary to train classifiers capable of distinguishing C_3_ from C_4_ species with >99% accuracy. The presence of distinguishing information here is in alignment with previous work suggesting positive selection acting on specific codons of *rbcL* in C_4_ species (Christin et al. 2008; Kapralov et al. 2011; Kapralov et al. 2012; Piot et al. 2018; Casola and Li 2022), but the extreme accuracy with which our RbcL models can classify PACMAD species as C_3_ or C_4_ further suggests a level of adherence to identified patterns of changes that are possibly critical for C_4_ evolution and/or maintenance.

Positions 101, 143, 281, 309, 418, and 468 of RbcL (all positions herein numbered relative to orthologous *Zea mays* sequences) were identified via RFE-CV as optimal features and were all that were necessary to inform a classifier with 99.98% accuracy. 101, 281, and 309 had previously been identified as strong candidates for being under positive selective pressure in C_4_ clades (Christin et al. 2008). Four hundred sixty-eight was later determined to be under the same selective pressure (Piot et al. 2018), and 143 had separately been identified as exhibiting convergent AA replacements in C_4_ clades (Casola and Li 2022). Three hundred and twenty-eight, although not one of the identified optimal features, had been one of the ten strongest selected features for our v2 model and this position had been highlighted in all three studies (Christin et al. 2008; Piot et al. 2018; Casola and Li 2022). Positive selection acting on residues 281 and 309 was not limited to grasses or even monocots. An Ala-281-Ser substitution and a Met-309-Ile substitution, the same substitutions distinguishing certain C_4_ species from ancestral C_3_ species in our dataset, were found to be positively selected for in C_4_ Amaranthaceae species as well (Kapralov et al. 2012). The Met-309-Ile substitution was again found to be positively selected for in C_4_ Flaveria species (Kapralov et al. 2011). This specific substitution was shown, in transplastomic experiments, to increase the carboxylation rate of Rubisco while reducing CO_2_ affinity (Whitney et al. 2011). Increased speed at the cost of CO_2_ affinity is a property associated with the C_4_-like enzyme due to the CO_2_ concentrating mechanisms employed by C_4_ species (Kapralov et al. 2011; Whitney et al. 2011). Although not discussed in the context of photosynthetic type, 143 and 262 (v2 model strongest feature) were identified as sites having undergone adaptive evolution in gymnosperms (Sen et al. 2011). Ser-143-Ala and Thr-262-Val substitutions were connected to ecological diversification while the ancestral state remained geographically localized (Sen et al. 2011). In our dataset, a similar polar-143-hydrophobic substitution (Thr-143-Ala) is exclusive to C_4_ species and arises in five distinct lineages. Structurally, 143, 262, 281, 309, 328, and 418 are all either surface exposed, interface proximal, and/or active site proximal residue positions (fig. 5) (Spreitzer and Salvucci 2002; Spreitzer et al. 2005; Sen et al. 2011). Substitutions at these key areas could alter complex stability, Rubisco holoenzyme expression, substrate binding efficiency, and/or regulation of Rubisco activation and therefore prove to be functionally relevant and possibly C_3_ and C_4_ distinguishing, mechanistically. Four hundred and eighteen which lies on an LSU–SSU interface was previously identified as part of a cluster that potentially plays a role in altering Rubisco's CO_2_/O_2_ specificity (Yu et al. 2005), a quality that differs between C_3_ and C_4_ species (Sage et al. 2012). This considerable alignment of identified optimal features with previously reported key sites associated with the evolution of C_4_ photosynthesis or related adaptive traits is both proof and an example of our framework's ability to arrive at biologically significant findings.

The *rbcL* gene has been the focus of the vast majority of comparative, plastid-encoded gene studies between C_3_ and C_4_ species. Selection tests, as discussed, have been employed to uncover sites in *rbcL* that are being acted upon by positive selective pressures in C_4_ lineages (Christin et al. 2008; Kapralov et al. 2011; Kapralov et al. 2012; Piot et al. 2018), but rarely have entire plastomes been screened for such sites. Piot et al. (2018) conducted one such plastome-wide screen in 113 PACMAD grasses and arrived at the conclusion that no obvious adaptive evolution occurred in protein-coding plastid genes, outside of *rbcL*, which could be attributed to the C_3_—C_4_ photosynthetic transition. Casola and Li (2022) more recently uncovered molecular convergence taking place in multiple chloroplast proteins via a comparative analysis of inferred AA replacements in C_3_ and C_4_ lineages, but from a more limited dataset of 64 PACMAD species. The results of our plastome wide screen, from 134 PACMAD species, do not attempt to dispute either finding because our framework is designed to answer different questions. While the aforementioned studies were designed to uncover positive selective pressures and convergence on individual amino acids independently of one another, our framework makes use of ML to consider all sites of a sequence of interest simultaneously. This allows for the detection of patterns or combinations of informative changes, across sites. Our work does demonstrate that plastid-encoded proteins, other than RbcL, harbor patterns of changes across PACMAD species that inform accurate classification models. These would be changes that are potentially associated with photosynthetic type. Sequences from NdhI, NdhA, RpoA, and NdhD were identified, in addition to RbcL, as being capable of informing classifiers with >90% accuracy.

During our investigations, we noted the enrichment of Ndh-subunit sequences among top informers of our v1, v2, and v3 models. This enrichment strongly implicates the NDH complex in roles related to, and likely differing between C_3_ and C_4_ metabolism. An increase in accumulation of the NDH complex in C_4_ species is suggested to aid in meeting the additional ATP demand required of C_4_ photosynthesis via NDH-mediated cyclic electron flow, an alternative to photosynthetic electron transport (Horváth et al. 2000; Martín and Sabater 2010; Nakamura et al. 2013; Ishikawa et al. 2016a, 2016b). While the complex is understood to play this pivotal role during photosynthesis few investigations into specific residue and substitution associations with C_4_ metabolism have taken place. We took advantage of our framework's ability to detect patterns across sites to determine if the combined plastid-encoded subunits of the NDH complex could inform accurate classification models and compared this performance to other plastidic complexes. Biologically relevant information that is lost when assessing individual subunits, such as functional redundancies and/or compensatory mutations among different subunits of a larger complex, is captured here by combining related subunits in our models. Our optimized NDH complex model had a classification accuracy of 97.07% and performed significantly better than the next best complex, ATP synthase, which informed an optimized model capable of 91.90% accuracy. Despite starting with the ten strongest features from all plastid-encoded Ndh subunits, after RFE-CV the final model made use of a combination of features from only NdhA, NdhD, NdhI, and NdhH, all of which inform a top-performing individual v1, v2, and/or v3 model. This again reinforces a potential biological relevance for these specific subunits, along with the entire complex, in the evolution and/or maintenance of C_4_ photosynthesis.

Residue 25 of NdhI exhibited the most notable level of parallel changes associated with the C_3_—C_4_ transition from identified NDH complex optimal features. NdhI exists as part of Subcomplex A (SubA) of the NDH complex as shown in recent structural work in *Arabidopsis thaliana* (PDB id: 7WFG) (Su et al. 2022). In this subcomplex (NdhH- NdhO), domains of NdhI are suggested to interact with ferredoxin (Fd) in the Fd-binding pocket (Laughlin et al. 2019; Zhang et al. 2020; Su et al. 2022), however residues near this binding site did not coincide with key sites identified by our framework. Instead, residue 25 of NdhI exists closer to the N-terminal end of this subunit, as part of a helical domain that is in contact with NdhT of Subcomplex E (SubE) (Yamamoto et al. 2011; Su et al. 2022). This contact between NdhT and SubA has been suggested to be necessary for the accumulation of SubA (Yamamoto et al. 2011) and shown to be strictly required for the assembly and functionality of the similarly structured human mitochondrial complex I (Stroud et al. 2016). Ishikawa et al. (2016a, 2016b) demonstrated that NDH accumulation is greatly increased in C_4_ species, relative to C_3_ species, and quite interestingly Nakamura et al. (2013) observed that accumulation of NdhH, a member of SubA, increased with the progression of C_4_ evolution (C_3_, C_3_–C_4_, C_4_-like, C_4_) in the genus *Flaveria*. Substitutions at NdhI-25 could therefore be altering NdhT-SubA interaction dynamics that are in turn corresponding to observed changes in accumulation levels of SubA and the NDH complex between C_3_ and C_4_ species. NdhT, however, does not just contact SubA at the NdhI interface. NdhT makes contact with NdhH, NdhI, NdhJ of SubA, and NdhA of SubM (Su et al. 2022). Features from NdhH and NdhA were identified as key sites from our NDH complex model, optimal features from NdhA informed the third best performing v3 model after RbcL and NdhI, and NdhH informed the sixth best performing v2 model. NdhH-269, an identified optimal feature informing our NDH complex model, is exposed on the same SubA face as NdhI-25 and is in close proximity to a possible NdhT–NdhH interface, as modeled in Su et al. (2022). Here, one can see how substitutions at various combinations of our identified key sites from NdhI, NdhA, or NdhH could similarly alter subunit interaction dynamics to potentially bring about the necessary increase in NDH accumulation for C_4_ photosynthesis (Ishikawa et al. 2016a, 2016b) and thus alleviate selective pressures at unchanged sites. This is an example of the kind of patterns of changes, associated with a convergent trait, which our ML framework can pick up on that might be overlooked with selection testing.

C_4_ species are often grouped according to the distinct biochemical pathways and Kranz anatomical variations they employ in the establishment of C_4_ photosynthesis (Hatch et al. 1975; Muhaidat et al. 2007). Three main C_4_ biochemical subtypes have been identified and they mainly differ in the mechanisms used for shuttling and decarboxylation of CO_2_. These subtypes are named according to the main decarboxlyation enzymes employed in each pathway, specifically, nicotinamide adenine dinucleotide phosphate (NADP)-malic enzyme (NADP-ME), nicotinamide adenine dinucleotide (NAD)-malic enzyme (NAD-ME), and phosphoenolpyruvate carboxykinase (PEP-CK) (Kanai and Edwards 1999). There are several more Kranz anatomy variations (Giussani et al. 2001; Muhaidat et al. 2007; Christin et al. 2008; Christin et al. 2009), or C_4_ anatomical subtypes, and it is expected that C_4_ subtypes likely differ in genotype-photosynthetic type associations due to the evolution of different biochemical and mechanistic approaches to establishing C_4_ photosynthesis. Variation in biochemical subtype is well represented within our PACMAD species dataset, however, less anatomical variation is present due to Poaceae C_4_ species largely employing Classical Kranz anatomy (supplementary table S1, Supplementary Material online) (Christin et al. 2009; Sage et al. 2011; Rudov et al. 2020). Supplementary Figure S4, Supplementary Material online summarizes the misclassification rates per C_4_ subtype of each of our identified top models in an attempt to determine if false classifications for certain models are associated with specific subtypes. Our RbcL v3 model only misclassified species of our dataset at a rate of 0.02%, and the only C_4_ species it would misclassify as C_3_, albeit rarely, was *Alloteropsis angusta*, a species with Neurachneoid Kranz-typing and NAD-ME + PEP-CK biochemical subtype. Apart from this, our RbcL model misclassified all C_4_ subtypes equally at a rate of 0%, suggesting that changes to RbcL are key to all C_4_ subtypes. Our NDH complex model misclassified NADP-ME, NAD-ME, and PEP-CK subtypes similarly at low rates of 4–6%, again suggesting that changes to the NDH complex, as a whole, and their associations with the evolution of C_4_ photosynthesis likely do not depend on biochemical subtype, or if dependance does exist, it is accounted for at the complex level. Other top v3 models (NdhI, NdhA, RpoA, and NdhD) showed variation in their rates of misclassification of the three biochemical subtypes. This suggests that these proteins, and changes to them, may play lesser or larger roles in C_4_ development depending on the evolved biochemical subtype. Misclassification based on Kranz-type did vary more obviously. Our NDH complex model misclassified Aristidoid Kranz-type species at a rate of 59.7%, Neurachneoid Kranz-type species at a rate of 33.1%, Classical Kranz-type species at a rate of 2.2%, and all other Kranz-types at 0%. This and similar variations in Kranz-type misclassification rates from other top models suggest that changes to certain plastid sequences might only be required for certain Kranz-typings, however, with the very limited representation of certain Kranz-types in our PACMAD dataset (e.g., only one Aristidoid species) it is difficult to effectively elaborate on such findings.

Several factors were considered in the implementation and validation of our models before attempting to draw conclusions from classification metrics. Importantly, it was necessary for our ML study to be designed in a manner that allowed for the deciphering of true C_3_/C_4_ associations from potential phylogenetic signals in the dataset. Our PACMAD species dataset, used for training and testing, encompasses a reported 16 independent origins of C_4_ photosynthesis (GPWG II, 2012) with intervening C_3_ lineages (fig. 1, supplementary table S1, Supplementary Material online), and this was to ensure the resulting classification metrics were a measure of C_3_ or C_4_ specific pattern detection instead of phylogenetic correlation. Cross validation with repeated (500 iterations) random subsampling (Dubitzky et al. 2007; Vabalas et al. 2019) was used for all models to generate average classification metrics for comparison that were not based solely on any one coincidentally optimal or suboptimal sampling of the dataset. Initial modeling on full-length sequences revealed great variation in informative power across the 76 protein sequences screened. This observed variation suggests a specificity when it comes to sequences capable of informing a strong classifier and this was important to our aim both for assigning biological significance and to again alleviate concerns of phylogenetic biases. Feature selection and RFE-CV were employed to generate models from a minimal number of features to avoid the potential for overfitting due to the feature number being much greater than the dataset size. RFE-CV was combined with permutation testing to ensure that coincident patterns found in randomly labeled sets could not inform classifiers with comparable performance metrics to true models. Length-accuracy, variation-accuracy, and MPD-accuracy correlation analyses (supplementary fig. S1, Supplementary Material online, fig. 3*D*) revealed only moderate associations present, meaning our optimized v3 models were not exclusively relying on the length or variation/noise present in sequence alignments to achieve strong classification metrics. We expect that if models were relying solely on phylogenetic signals to distinguish C_3_ from C_4_ species then sequences of greater length, variation, and/or MPD should typically inform better classifiers due to greater opportunity to pick up on phylogenetic-specific information. RbcL and NdhI were neither among the most variable nor lengthy sequences of all considered, yet they informed the two of the strongest performing v1, v2, and v3 models. However, we do acknowledge that RpoC2, MatK, and NdhF were among the lengthiest and most variable sequences of all queried and so for this reason we did not attempt to draw conclusions from the relatively strong performance of their corresponding models. A smaller study to identify optimal features/residues from eudicot species was conducted, using our pipeline, and summarized in supplementary table S3, Supplementary Material online and supplementary table S4, Supplementary Material online. Despite the species of our eudicot dataset being distant relatives to grasses, several identified key residues for distinguishing C_3_/C_4_ species in eudicots overlapped with those we identified as key from PACMAD sequences. The identification of overlapping key residue combinations in species far removed from those of the PACMAD clade is a further indication that our study is identifying true C_3_/C_4_ distinguishing signals. Complete agreement on key residues identified from eudicots and grasses is not to be expected because of the differences in Kranz-types and biochemical types between the C_4_ species of these two groups (supplementary table S1, Supplementary Material online, supplementary table S3, Supplementary Material online). Furthermore, the current limited eudicot sequence data availability does not allow for as robust of a analysis as was carried out with our PACMAD dataset. C_3_ and C_4_ distinguishing information found in eudicot plastomes, and/or other specific families with several C_4_ origins, will be very interesting to explore further, as soon as relevant sequence data becomes available. The same potential for discovery and validation of key residue combinations in C_4_ nuclear markers, such as phosphoenolpyruvate carboxylase and pyruvate, phosphate dikinase, exists with our pipeline, again, if and when sufficient sequence data becomes available.

Countless research efforts today are geared toward contributing to a greater understanding of the C_4_ pathway, not least because of the obvious potential for applications in synthetic biology and crop improvement (Matsuoka et al. 2001; Sage and Sage 2009; Kajala et al. 2011; Schuler et al. 2016; Jobe et al. 2020; Roell et al. 2021). The C_4_ trait is a formidable one because it has allowed inheriting species to outperform the ancestral C_3_ state in hot and arid regions due to a convergence of several biochemical, physiological, and anatomical features with adaptive significance (Still et al. 2003; Sage et al. 2012; Atkinson et al. 2016). The identification of sequence-photosynthetic type associations in grasses, a family to which many of the world's staple crops belong, would be key to furthering an understanding of what might be necessary to emulate C_4_ efficiency in crop species. The presented work is a demonstration and validation of the use of an ML framework in offering an alternative perspective from which to determine key sequences and sites, from across grass plastomes, potentially driving the evolution of this complex, convergent trait.

## Materials and Methods

### Dataset Assembly

Complete plastomes for 135 Poaceae species (134 PACMAD + *Anomochloa marantoidea* for rooting) were retrieved from the Chloroplast Genome Database (CpGDB) (Singh et al. 2020). Fifty-nine C_3_ species and 76 C_4_ species (supplementary table S1, Supplementary Material online) were selected based on the availability of experimental verification of photosynthetic type as summarized in Osborne et al. (2014) and used in Piot et al. (2018). Species sampling spanned multiple reported C_4_ origins (GPWG II 2012) from the PACMAD clade and was curated specifically to include C_3_ and C_4_ species more closely related to each other than to other species of the same photosynthetic type in the dataset. C_4_ species were sampled from 16 different lineages with independent C_4_ origins (fig. 1) and C_3_ species were sampled from closely related lineages of each.

Protein-encoding regions were extracted from each plastome according to gene coordinates obtained from the CpGDB. Each of 76 plastid-encoded coding sequences from across 135 Poaceae species was translated using the plant plastid code (translation table 11) to obtain AA sequences.

### Phylogenetic Analysis

Sequence alignments of nine plastid marker genes (*rbcL, ndhF, matK, rpoC2, psaA, psaB, psaI, rpl32,* and *ccsA*) from 134 PACMAD species of our dataset was performed using MUltiple Sequence Comparison by Log-Expectation (Edgar 2004) and concatenated. Maximum likelihood phylogenies were estimated from the concatenated alignments using RAxML (GTR+Γ model) (Stamatakis 2014) with 1,000 bootstrap replicates and rooted to *Anomochloa marantoidea*, a grass species existing outside of the PACMAD clade.

### Feature Engineering From Alignment Data

Feature lists were created for sets of sequence alignment data from each of 76 plastid-encoded protein sequences of the 134 PACMAD species of our dataset (see Data Availability). Here we vectorize each of our sets of alignment data to obtain a list of features corresponding to each position in each alignment for entire sequence interpretability by our ML algorithms. To do this, we created keys for each position in each alignment that map corresponding residues/gaps to a value up to N–1 where N is the number of different characters (AA or gap) that appear at each position. For the vast majority of positions in each alignment, *N* <= 2 and therefore each AA at each position is typically represented by [0] or [1]. However, a small portion of amino acids are represented by [2], [3], or greater if *N* > 2 at any position. We acknowledge that it is inadvisable to represent nonlinearly related variables as such but combining the small frequency of occurrence with a relatively large number of features makes the negative effects on the overall classifier near negligible while still extracting potentially distinguishing information from highly variable positions. To demonstrate this, we trained models with oppositely swapped AA encodings (with respect to our initial feature representation set) for 50% random positions, and randomly swapped AA encodings at each alignment position and compared these to models trained on our initial feature representation set (supplementary fig. S5, Supplementary Material online). Identification of top classifiers and most informative residues, after recursive feature elimination, remained the same regardless of the specific AA encoding at each alignment position. Each sequence represented as a list of predominantly binary features, was labeled as C_3_ or C_4_ in accordance with the specific species/plastome it had been extracted from.

### C_3_/C_4_ Classification Modelling With Cross Validation

The following was achieved in Python using tools available with the Scikit-learn library (Pedregosa et al. 2011). For each protein sequence, labeled (C_3_/C_4_) feature data representing the sequence alignment datasets of the curated 135 Poaceae species were split into training and independent test sets (70:30 split). Each feature was standardized and the labeled, standardized feature data from the training sets were used for supervised learning with a logistic regression classification algorithm.

Repeated random subsampling (Dubitzky et al. 2007), also known as Nested Cross-Validation, was used for cross-validation of each model as described in Vabalas et al. 2019. Five hundred different models were generated based on 500 different and random 70:30 splits into training and test sets respectively for each sequence dataset while keeping all other parameters equal. Each of these 500 models was independent of one another and each set of test data was kept independent from the training of their respective model to prevent data leakage. The average accuracy across 500 models was computed and these average accuracies are what we compare in this study. Classification metrics (AUROC, precision, recall, F1) were also computed as averages after cross validation. Models built on randomized labels were generated, for comparison, by providing the logistic regression classification algorithm with randomly labeled (C_3_/C_4_), but in equal proportion, feature data.

### Feature Selection and Recursive Feature Elimination

Initial logistic regression models (v1 models, see fig. 1) were parsed, and the regression coefficients attributed to each feature in the final models were extracted. Specifically for regression coefficient parsing and extraction, entire datasets, instead of split datasets, were used. The ten strongest features, for use in our v2 models, were the positions corresponding to the ten largest (absolute) coefficient values of the final decision function. The v2 models were generated, as described above, using only the identified ten strongest features from the corresponding v1 models.

To determine specific feature combinations from each feature set that informed top-performing classification models we employed recursive feature elimination (Guyon et al. 2002) with cross validation (RFE-CV). Starting with all initial features, 500 different logistic regression models would be generated using 500 different and random 70:30 splits into training and test sets, respectively, and averages for all classification metrics would be recorded. The most consistent weakest feature—lowest (absolute) attributed regression coefficient—from across 500 samplings would be eliminated from the subsequent feature set and the process repeated until one feature remains. We employed a custom implementation of RFE-CV that allowed recursion to continue past the point of no improvement in accuracy so that we were always able to determine performance for feature sets of ten or fewer features. The strongest performing feature combination of ten or fewer features was extracted as the optimal feature set. This generates classification metric data, after cross validation, for as many iteratively stronger combinations of features as there were initial features.

For the comparative analysis of informative information held by plastid-encoded genes and the identification of potential key residues informing C_3_/C_4_ status, we made use of our entire PACMAD dataset (134 species) for optimal feature selection via RFE-CV. This was done in order to screen as much sequence information as possible for potentially informative residues. Supplementary Table S5, Supplementary Material online summarizes the results of conducting RFE-CV with 90% of our PACMAD dataset and testing models trained with optimal features on an independent validation set (10% of our PACMAD dataset).

### Accuracy Correlation Analyses

Mean pairwise distance was calculated as a measure of the total variation across the various lengths of our sequence alignments (Shpak et al. 2017). For each alignment, the average number of differences between each possible pair of sequences in a set was computed and then plotted against the respective classification model's accuracy. For variation-accuracy correlation analyses, MPD as a proportion of sequence length (per 100 residues) was calculated. A length-accuracy correlation plot was also generated by plotting model accuracy against respective sequence alignment lengths.

### Combined Subunit/Complex Classification Modeling

For each complex queried (table 2), feature selection was used to obtain the ten strongest features from each of its constituent subunits as listed in supplementary table S2, Supplementary Material online. The optimal features from constituent subunits were concatenated and RFE-CV was employed to obtain classification metrics from iteratively stronger combinations of features across subunits. The strongest performing models, of iterations making use of ten or fewer features, from each complex were compared in our study. Recent work has revealed that PsbN might not be a constituent member of the PSII supercomplex (Torabi et al. 2014) and so was not included in our PSII model development. Rpl32 was not included in our 50S model because of its absence from several Poaceae plastid genomes.

## Supplementary Material

evad129_Supplementary_Data

## Data Availability

The data underlying this article are available in the article and its online Supplementary material. All sequence alignment data informing components of this study are openly available on figshare at https://doi.org/10.6084/m9.figshare.21379725.v1 and the necessary code for pipeline implementation is publically available at https://github.com/Nil29/C4PredictorAnalysis
