# Supplementary material for: A Machine Learning Framework Identifies Plastid-Encoded Proteins Harboring C3 and C4 Distinguishing Sequence Information
Source: Genome Biol Evol. 2023 Jul 18;15(7):evad129. doi: 10.1093/gbe/evad129 (PMC10368328; doi:10.1093/gbe/evad129)
Supplement: evad129_Supplementary_Data [file evad129_supplementary_data.docx]

**Table S1.** List of Poaceae species in assembled dataset used in this study. C_3_/C_4_ status, Kranz-type, and Biochemical type are determined as referenced in Sage et al. (2011) and from Giussani et al. (2001) , Muhaidat et al. (2007), Christin et al. (2008), Christin et al. (2009), Edwards and Vosnesenskya (2011), GPWG II (2012), Osborne et al. (2014), Piot et al. (2018), and Rudov et al. (2020). Typing from latest study is used for conflicting reports.

| **Subfamily** | **Species** | **C3/C4** | **C4 lineage** | **Kranz type** | **Biochemical type** |
| --- | --- | --- | --- | --- | --- |
| Anomochlooideae | *Anomochloa marantoidea* | C3 |  |  |  |
| Aristidoideae | *Sartidia dewinteri* | C3 |  |  |  |
| Aristidoideae | *Sartidia isaloensis* | C3 |  |  |  |
| Aristidoideae | *Sartidia perrieri* | C3 |  |  |  |
| Aristidoideae | *Aristida rufescens* | C4 | *Aristida* | Aristidoid | NADP-ME |
| Aristidoideae | *Stipagrostis hirtigluma* | C4 | *Stipagrostis* | Stipagrostroid | NADP-ME |
| Arundinoideae | *Amphipogon turbinatus* | C3 |  |  |  |
| Arundinoideae | *Crinipes longifolius* | C3 |  |  |  |
| Arundinoideae | *Dregeochloa pumilla* | C3 |  |  |  |
| Arundinoideae | *Elytrophorus globularis* | C3 |  |  |  |
| Arundinoideae | *Molinia caerulea* | C3 |  |  |  |
| Arundinoideae | *Phragmites australis* | C3 |  |  |  |
| Arundinoideae | *Styppeiochloa gynoglossa* | C3 |  |  |  |
| Arundinoideae | *Amphipogon caricinus* | C3 |  |  |  |
| Arundinoideae | *Crinipes abyssinicus* | C3 |  |  |  |
| Arundinoideae | *Dichaetaria wightii* | C3 |  |  |  |
| Arundinoideae | *Elytrophorus spicatus* | C3 |  |  |  |
| Arundinoideae | *Hakonechloa macra* | C3 |  |  |  |
| Arundinoideae | *Monachather paradoxus* | C3 |  |  |  |
| Chloridoideae | *Aeluropus lagopoides* | C4 | Chloridoideae | Classical | NAD-ME, PCK |
| Chloridoideae | *Astrebla pectinata* | C4 | Chloridoideae | Classical | NAD-ME |
| Chloridoideae | *Bouteloua curtipendula* | C4 | Chloridoideae | Classical | NAD-ME, PCK |
| Chloridoideae | *Bouteloua gracilis* | C4 | Chloridoideae | Classical | NAD-ME |
| Chloridoideae | *Centropodia glauca* | C4 | Centropodia | Classical | NAD-ME |
| Chloridoideae | *Chloris barbata* | C4 | Chloridoideae | Classical | NAD-ME, PCK |
| Chloridoideae | *Chloris virgata* | C4 | Chloridoideae | Classical | NAD-ME, PCK |
| Chloridoideae | *Cynodon dactylon* | C4 | Chloridoideae | Classical | NAD-ME |
| Chloridoideae | *Dactyloctenium aegyptium* | C4 | Chloridoideae | Classical | NAD-ME |
| Chloridoideae | *Eleusine indica* | C4 | Chloridoideae | Classical | NAD-ME |
| Chloridoideae | *Enneapogon caerulescens* | C4 | Chloridoideae | Classical | NAD-ME, PCK |
| Chloridoideae | *Enneapogon oblongus* | C4 | Chloridoideae | Classical | NAD-ME, PCK |
| Chloridoideae | *Halopyrum mucronatum* | C4 | Chloridoideae | Classical | NAD-ME, PCK |
| Chloridoideae | *Leptochloa virgata* | C4 | Chloridoideae | Classical | NAD-ME, PCK |
| Chloridoideae | *Melanocenchris abyssinica* | C4 | Chloridoideae | Classical | NAD-ME, PCK |
| Chloridoideae | *Orinus kokonoricus* | C4 | Chloridoideae | Classical | NAD-ME, PCK |
| Chloridoideae | *Oropetium aristatum* | C4 | Chloridoideae | Classical | NAD-ME, PCK |
| Chloridoideae | *Oxychloris scariosa* | C4 | Chloridoideae | Classical | NAD-ME, PCK |
| Chloridoideae | *Distichlis bajaensis* | C4 | Chloridoideae | Classical | NAD-ME |
| Danthonioideae | *Alloeochaete namuliensis* | C3 |  |  |  |
| Danthonioideae | *Alloeochaete oreogena* | C3 |  |  |  |
| Danthonioideae | *Alloeochaete uluguruensis* | C3 |  |  |  |
| Danthonioideae | *Chionochloa macra* | C3 |  |  |  |
| Danthonioideae | *Danthonia californica* | C3 |  |  |  |
| Danthonioideae | *Merxmuellera tsaratananensis* | C3 |  |  |  |
| Danthonioideae | *Rytidosperma pallidum* | C3 |  |  |  |
| Danthonioideae | *Rytidosperma semiannulare* | C3 |  |  |  |
| Danthonioideae | *Schismus arabicus* | C3 |  |  |  |
| Danthonioideae | *Tenaxia guillarmodiae* | C3 |  |  |  |
| Danthonioideae | *Tribolium hispidum* | C3 |  |  |  |
| Danthonioideae | *Cortaderia selloana* | C3 |  |  |  |
| Danthonioideae | *Phaenanthoecium koestlinii* | C3 |  |  |  |
| Micrairoideae | *Coelachne africana* | C3 |  |  |  |
| Micrairoideae | *Isachne albens* | C3 |  |  |  |
| Micrairoideae | *Isachne distichophylla* | C3 |  |  |  |
| Micrairoideae | *Limnopoa meeboldii* | C3 |  |  |  |
| Micrairoideae | *Eriachne aristidea* | C4 | Eriachne | Eriachneoid | NADP-ME |
| Micrairoideae | *Eriachne mucronata* | C4 | Eriachne | Eriachneoid | NADP-ME |
| Micrairoideae | *Eriachne obtusa* | C4 | Eriachne | Eriachneoid | NADP-ME |
| Micrairoideae | *Eriachne pallescens* | C4 | Eriachne | Eriachneoid | NADP-ME |
| Micrairoideae | *Pheidochloa gracilis* | C4 | Eriachne | Eriachneoid | NADP-ME |
| Micrairoideae | *Eriachne stipacea* | C4 | Eriachne | Eriachneoid | NADP-ME |
| Panicoideae | *Amphicarpum muhlenbergianum* | C3 |  |  |  |
| Panicoideae | *Homolepis isocalycia* | C3 |  |  |  |
| Panicoideae | *Otachyrium versicolor* | C3 |  |  |  |
| Panicoideae | *Sacciolepis indica* | C3 |  |  |  |
| Panicoideae | *Thyridolepis xerophila* | C3 |  |  |  |
| Panicoideae | *Thysanolaena latifolia* | C3 |  |  |  |
| Panicoideae | *Zeugites pittieri* | C3 |  |  |  |
| Panicoideae | *Centotheca lappacea* | C3 |  |  |  |
| Panicoideae | *Chasmanthium laxum* | C3 |  |  |  |
| Panicoideae | *Entolasia imbricata* | C3 |  |  |  |
| Panicoideae | *Gynerium sagittatum* | C3 |  |  |  |
| Panicoideae | *Lasiacis nigra* | C3 |  |  |  |
| Panicoideae | *Lecomtella madagascariensis* | C3 |  |  |  |
| Panicoideae | *Oplismenus hirtellus* | C3 |  |  |  |
| Panicoideae | *Plagiantha tenella* | C3 |  |  |  |
| Panicoideae | *Brachiaria fragrans* | C3 |  |  |  |
| Panicoideae | *Chasechloa egregia* | C3 |  |  |  |
| Panicoideae | *Chasechloa madagascariensis* | C3 |  |  |  |
| Panicoideae | *Homolepis aturensis* | C3 |  |  |  |
| Panicoideae | *Streptostachys asperifolia* | C3 |  |  |  |
| Panicoideae | *Pseudolasiacis leptolomoides* | C3 |  |  |  |
| Panicoideae | *Steinchisma laxum* | C3 |  |  |  |
| Panicoideae | *Chandrasekharania keralensis* | C3 |  |  |  |
| Panicoideae | *Jansenella griffithiana* | C3 |  |  |  |
| Panicoideae | *Jansenella neglecta* | C3 |  |  |  |
| Panicoideae | *Alloteropsis semialata* | C4 | *Alloteropsis* | Neurachneoid | NAD-ME, PCK |
| Panicoideae | *Alloteropsis angusta* | C4 | *Alloteropsis* | Neurachneoid | NAD-ME, PCK |
| Panicoideae | *Alloteropsis cimicina* | C4 | *Alloteropsis* | Neurachneoid | NAD-ME, PCK |
| Panicoideae | *Alloteropsis paniculata* | C4 | *Alloteropsis* | Neurachneoid | NAD-ME, PCK |
| Panicoideae | *Zea mays* | C4 | Andropogoneae | Classical | NADP-ME |
| Panicoideae | *Andropogon distachyos* | C4 | Andropogoneae | Classical | NADP-ME |
| Panicoideae | *Apluda mutica* | C4 | Andropogoneae | Classical | NADP-ME |
| Panicoideae | *Arthraxon hispidus* | C4 | Andropogoneae | Classical | NADP-ME |
| Panicoideae | *Chrysopogon gryllus* | C4 | Andropogoneae | Classical | NADP-ME |
| Panicoideae | *Coix lacryma-jobi* | C4 | Andropogoneae | Classical | NADP-ME |
| Panicoideae | *Cymbopogon citratus* | C4 | Andropogoneae | Classical | NADP-ME |
| Panicoideae | *Dichanthium annulatum* | C4 | Andropogoneae | Classical | NADP-ME |
| Panicoideae | *Dichanthium sericeum* | C4 | Andropogoneae | Classical | NADP-ME |
| Panicoideae | *Dimeria ornithopoda* | C4 | Andropogoneae | Classical | NADP-ME |
| Panicoideae | *Eulaliopsis binata* | C4 | Andropogoneae | Classical | NADP-ME |
| Panicoideae | *Heteropogon contortus* | C4 | Andropogoneae | Classical | NADP-ME |
| Panicoideae | *Heteropogon triticeus* | C4 | Andropogoneae | Classical | NADP-ME |
| Panicoideae | *Imperata cylindrica* | C4 | Andropogoneae | Classical | NADP-ME |
| Panicoideae | *Miscanthus sinensis* | C4 | Andropogoneae | Classical | NADP-ME |
| Panicoideae | *Miscanthus sacchariflorus* | C4 | Andropogoneae | Classical | NADP-ME |
| Panicoideae | *Pogonatherum paniceum* | C4 | Andropogoneae | Classical | NADP-ME |
| Panicoideae | *Pseudosorghum fasciculare* | C4 | Andropogoneae | Classical | NADP-ME |
| Panicoideae | *Saccharum officinarum* | C4 | Andropogoneae | Classical | NADP-ME |
| Panicoideae | *Saccharum spontaneum* | C4 | Andropogoneae | Classical | NADP-ME |
| Panicoideae | *Axonopus fissifolius* | C4 | *Axonopus* | Classical | NADP-ME |
| Panicoideae | *Axonopus ramosus* | C4 | *Axonopus* | Classical | NADP-ME |
| Panicoideae | *Coleataenia prionitis* | C4 | *Coleataenia* | Neurachneoid | NADP-ME |
| Panicoideae | *Digitaria exilis* | C4 | *Digitaria* | Classical | NADP-ME |
| Panicoideae | *Echinochloa colona* | C4 | Echinochloa | Classical | NADP-ME |
| Panicoideae | *Echinochloa crus-galli* | C4 | Echinochloa | Classical | NADP-ME |
| Panicoideae | *Echinochloa esculenta* | C4 | *Echinochloa* | Classical | NADP-ME |
| Panicoideae | *Echinochloa frumentacea* | C4 | *Echinochloa* | Classical | NADP-ME |
| Panicoideae | *Echinochloa stagnina* | C4 | *Echinochloa* | Classical | NADP-ME |
| Panicoideae | *Panicum capillare* | C4 | MPC | Classical | NAD-ME |
| Panicoideae | *Panicum miliaceum* | C4 | MPC | Classical | NAD-ME |
| Panicoideae | *Paspalidium geminatum* | C4 | MPC | Classical | NADP-ME |
| Panicoideae | *Megathyrsus maximus* | C4 | MPC | Classical | NAD-ME, PCK |
| Panicoideae | *Eriochloa meyeriana* | C4 | MPC | Classical | NAD-ME, PCK |
| Panicoideae | *Setaria italica* | C4 | MPC | Classical | NADP-ME |
| Panicoideae | *Setaria viridis* | C4 | MPC | Classical | NADP-ME |
| Panicoideae | *Stenotaphrum secundatum* | C4 | MPC | Classical | NADP-ME |
| Panicoideae | *Tricholaena monachne* | C4 | MPC | Classical | NAD-ME, PCK |
| Panicoideae | *Oncorachis ramosa* | C4 | *Oncorachis* | Classical | NADP-ME |
| Panicoideae | *Paraneurachne muelleri* | C4 | *Paraneurachne* | Neurachneoid | NADP-ME, PCK |
| Panicoideae | *Paspalum dilatatum* | C4 | *Paspalum* | Classical | NADP-ME |
| Panicoideae | *Paspalum paniculatum* | C4 | *Paspalum* | Classical | NADP-ME |
| Panicoideae | *Paspalum vaginatum* | C4 | *Paspalum* | Classical | NADP-ME |
| Panicoideae | *Danthoniopsis dinteri* | C4 | Tristachyideae | Arundinelloid | NADP-ME |
| Panicoideae | *Tristachya humbertii* | C4 | Tristachyideae | Arundinelloid | NADP-ME |

MPC - Melinidinae + Panicinae + Cenchrinae.

**Table S2.** Summary of v2 model performances after ten strongest feature selection

| **AA seq** | **Ten Strongest Features (ap)** | **Residue Positions (*Zm*)** | **Acc** |
| --- | --- | --- | --- |
| atpA | 456, 503, 488, 215, 467, 347, 460, 383, 97, 10 | 456, 503, 488, 215, 467, 347, 460, 383, 97, 10 | **0.8019** |
| atpB | 96, 105, 4, 497, 386, 496, 450, 383, 495, 382 | 96, 105, 4, 497, 386, 496, 450, 383, 495, 382 | **0.8798** |
| atpE | 106, 121, 55, 102, 130, 16, 25, 62, 113, 61 | 106, 121, 55, 102, 130, 16, 25, 62, 113, 61 | **0.8251** |
| atpF | 36, 73, 70, 113, 14, 135, 155, 128, 98, 188 | 36, 68, 65, 108, 14, 130, 150, 123, 93, 183 | **0.7533** |
| atpH | 11, 4, 44, 7, 79, 78, 77, 76, 75, 74 | 11, 4, 44, 7, 79, 78, 77, 76, 75, 74 | **0.5461** |
| atpI | 92, 212, 102, 26, 75, 52, 57, 59, 10, 67 | 92, 212, 102, 26, 75, 52, 57, 59, 10, 67 | **0.669** |
| ccsA | 100, 170, 296, 183, 213, 328, 195, 173, 203, 329 | 100, 168, 287, 178, 204, 319, 186, 171, 194, 319 | **0.8748** |
| clpP | 137, 138, 16, 220, 60, 135, 212, 59, 141, 199 | 137, 138, 16, 217, 60, 135, 209, 59, 141, 199 | **0.6213** |
| cemA | 88, 138, 133, 45, 112, 190, 46, 70, 34, 44 | 84, 132, 127, 41, 106, 184, 42, 66, 30, 40 | **0.6906** |
| infA | 17, 109, 55, 104, 22, 30, 56, 120, 16, 112 | 7, 95, 41, 90, 8, 16, 42, 106, 6, 98 | **0.7094** |
| matK | 66, 314, 246, 147, 436, 49, 378, 191, 226, 416 | 33, 274, 206, 111, 396, 16, 338, 153, 188, 376 | **0.8913** |
| ndhA | 36, 110, 5, 319, 9, 26, 301, 294, 141, 25 | 34, 108, 5, 314, 7, 24, 296, 289, 139, 23 | **0.8479** |
| ndhB | 28, 290, 514, 347, 483, 246, 133, 497, 156, 496 | 28, 279, 503, 336, 472, 246, 133, 486, 156, 485 | **0.7937** |
| ndhC | 9, 106, 5, 29, 27, 120, 36, 105, 30, 35 | 9, 106, 5, 29, 27, 120, 36, 105, 30, 35 | **0.6739** |
| ndhD | 114, 501, 497, 376, 218, 64, 109, 76, 451, 442 | 112, 499, 495, 374, 216, 62, 107, 74, 449, 440 | **0.8319** |
| ndhE | 2, 50, 97, 5, 100, 63, 18, 27, 81, 85 | 2, 50, 97, 5, 100, 63, 18, 27, 81, 85 | **0.6253** |
| ndhF | 89, 56, 340, 540, 669, 602, 336, 583, 560, 383 | 89, 56, 340, 529, 654, 591, 336, 572, 549, 383 | **0.669** |
| ndhG | 104, 90, 101, 75, 16, 173, 102, 44, 13, 136 | 104, 90, 101, 75, 16, 173, 102, 44, 13, 136 | **0.8315** |
| ndhH | 23, 270, 277, 43, 274, 48, 254, 200, 17, 12 | 18, 265, 272, 38, 269, 43, 249, 195, 12, 7 | **0.8627** |
| ndhI | 25, 5, 147, 89, 153, 49, 91, 84, 88, 117 | 25, 5, 147, 89, 153, 49, 91, 84, 88, 117 | **0.9317** |
| ndhJ | 91, 27, 34, 3, 14, 124, 119, 67, 80, 159 | 91, 27, 34, 3, 14, 124, 119, 67, 80, 159 | **0.6935** |
| ndhK | 72, 22, 8, 156, 10, 94, 48, 13, 32, 37 | 50, 0, 0, 134, 0, 72, 26, 0, 10, 15 | **0.7052** |
| petA | 20, 63, 11, 5, 290, 26, 145, 177, 14, 32 | 20, 63, 11, 5, 290, 26, 145, 177, 14, 32 | **0.7595** |
| petB | 144, 19, 138, 14, 179, 82, 142, 25, 223, 162 | 144, 19, 138, 14, 179, 82, 142, 25, 215, 162 | **0.6295** |
| petD | 150, 156, 154, 169, 64, 71, 31, 147, 67, 17 | 135, 141, 139, 154, 49, 56, 16, 132, 52, 3 | **0.5289** |
| petG | 25, 12, 36, 35, 34, 33, 32, 31, 30, 29 | 25, 12, 36, 35, 34, 33, 32, 31, 30, 29 | **0.5552** |
| petL | 29, 19, 24, 4, 25, 7, 20, 21, 26, 30 | 29, 19, 24, 4, 25, 7, 20, 21, 26, 30 | **0.6317** |
| petN | 5, 12, 18, 6, 9, 31, 30, 2, 1, 29 | 3, 10, 16, 4, 7, 29, 28, 0, 0, 27 | **0.556** |
| psaA | 274, 34, 484, 310, 427, 6, 144, 677, 80, 625 | 274, 34, 484, 310, 427, 6, 144, 677, 80, 625 | **0.821** |
| psaB | 224, 129, 492, 152, 483, 216, 502, 151, 248, 405 | 224, 129, 492, 152, 483, 216, 502, 151, 248, 405 | **0.6337** |
| psaC | 29, 70, 16, 71, 81, 80, 79, 78, 77, 76 | 29, 70, 16, 71, 81, 80, 79, 78, 77, 76 | **0.6204** |
| psaI | 24, 4, 30, 28, 5, 21, 34, 33, 22, 6 | 24, 4, 30, 28, 5, 21, 32, 32, 22, 6 | **0.7658** |
| psaJ | 4, 21, 41, 40, 39, 38, 37, 36, 35, 34 | 4, 21, 41, 40, 39, 38, 37, 36, 35, 34 | **0.5452** |
| psbA | 230, 235, 345, 155, 231, 349, 346, 7, 348, 264 | 230, 235, 345, 155, 231, 349, 346, 7, 348, 264 | **0.6245** |
| psbB | 182, 124, 73, 79, 410, 121, 290, 71, 296, 345 | 182, 124, 73, 79, 410, 121, 290, 71, 296, 345 | **0.7176** |
| psbC | 1, 441, 191, 291, 221, 2, 39, 451, 193, 259 | 0, 427, 177, 277, 207, 0, 25, 437, 179, 245 | **0.842** |
| psbD | 5, 9, 149, 8, 18, 155, 122, 146, 288, 4 | 5, 9, 149, 8, 18, 155, 122, 146, 288, 4 | **0.5899** |
| psbE | 79, 71, 59, 74, 78, 81, 21, 68, 83, 82 | 79, 71, 59, 74, 78, 81, 21, 68, 83, 82 | **0.5247** |
| psbF | 31, 18, 40, 5, 39, 38, 37, 36, 35, 34 | 30, 17, 39, 4, 38, 37, 36, 35, 34, 33 | **0.5769** |
| psbH | 11, 72, 13, 21, 28, 15, 42, 45, 71, 46 | 11, 72, 13, 21, 28, 15, 42, 45, 71, 46 | **0.7643** |
| psbI | 52, 22, 15, 14, 13, 12, 9, 8, 7, 6 | N/A | **0.5416** |
| psbJ | 27, 24, 32, 46, 34, 30, 44, 43, 42, 41 | 20, 17, 25, 39, 27, 23, 37, 36, 35, 34 | **0.7423** |
| psbK | 25, 47, 8, 41, 12, 21, 17, 44, 23, 4 | 23, 45, 8, 39, 12, 19, 17, 42, 21, 4 | **0.7235** |
| psbL | 12, 13, 10, 38, 37, 36, 35, 34, 33, 32 | 12, 13, 10, 38, 37, 36, 35, 34, 33, 32 | **0.5495** |
| psbM | 17, 33, 30, 24, 5, 15, 12, 25, 20, 32 | 17, 33, 30, 24, 5, 15, 12, 25, 20, 32 | **0.6144** |
| psbN | 7, 10, 43, 42, 41, 40, 39, 38, 37, 36 | 7, 10, 43, 42, 41, 40, 39, 38, 37, 36 | **0.541** |
| psbT | 28, 35, 34, 33, 29, 32, 31 | 25, 32, 31, 30, 26, 29, 28 | **0.5371** |
| psbZ | 28, 48, 17, 15, 22, 32, 31, 30, 10, 69 | 20, 40, 9, 7, 14, 24, 23, 22, 2, 61 | **0.5902** |
| rbcL | 281, 468, 143, 328, 262, 228, 418, 101, 309, 270 | 281, 468, 143, 328, 262, 228, 418, 101, 309, 270 | **0.992** |
| rpl2 | 175, 42, 185, 266, 25, 104, 19, 164, 152, 280 | 169, 38, 179, 260, 21, 100, 15, 158, 146, 273 | **0.8455** |
| rpl14 | 67, 50, 46, 35, 59, 109, 9, 41, 111, 58 | 67, 50, 46, 35, 59, 109, 9, 41, 111, 58 | **0.5972** |
| rpl16 | 120, 142, 29, 139, 33, 83, 25, 126, 116, 71 | 113, 135, 22, 132, 26, 76, 18, 119, 109, 64 | **0.7051** |
| rpl20 | 109, 124, 75, 17, 74, 12, 118, 92, 93, 9 | 103, 118, 74, 17, 73, 12, 112, 86, 87, 9 | **0.7643** |
| rpl22 | 103, 137, 98, 10, 38, 114, 141, 75, 131, 128 | 102, 136, 97, 10, 37, 113, 140, 74, 130, 127 | **0.7433** |
| rpl23 | 92, 67, 62, 37, 63, 70, 73, 71, 28, 81 | 92, 67, 62, 37, 63, 70, 73, 71, 28, 81 | **0.6662** |
| rpl32 | 58, 19, 57, 56, 46, 39, 42, 10, 59, 8 | 56, 19, 55, 54, 46, 39, 42, 10, 57, 8 | **0.6992** |
| rpl33 | 4, 22, 35, 10, 2, 62, 26, 28, 54, 43 | 4, 22, 35, 10, 2, 62, 26, 28, 54, 43 | **0.6564** |
| rpl36 | 20, 16, 18, 9, 22, 25, 37, 36, 35, 34 | 20, 16, 18, 9, 22, 25, 37, 36, 35, 34 | **0.5593** |
| rps2 | 67, 226, 232, 181, 231, 146, 74, 132, 32, 33 | 67, 226, 232, 181, 231, 146, 74, 132, 32, 33 | **0.6388** |
| rps3 | 118, 179, 197, 74, 110, 174, 28, 182, 109, 127 | 105, 166, 184, 61, 97, 161, 28, 169, 96, 114 | **0.7721** |
| rps4 | 151, 71, 148, 77, 176, 158, 17, 23, 155, 111 | 151, 71, 148, 77, 176, 158, 17, 23, 155, 111 | **0.7138** |
| rps7 | 121, 2, 29, 115, 7, 142, 132, 144, 80, 40 | 121, 2, 29, 115, 7, 142, 132, 144, 80, 40 | **0.614** |
| rps8 | 19, 67, 114, 54, 80, 68, 126, 125, 34, 77 | 19, 67, 114, 54, 80, 68, 126, 125, 34, 77 | **0.8064** |
| rps11 | 13, 59, 85, 12, 14, 57, 9, 122, 17, 115 | 13, 59, 85, 12, 14, 57, 9, 122, 17, 115 | **0.7918** |
| rps12 | 16, 134, 21, 19, 137, 140, 139, 27, 138, 136 | 14, 119, 19, 17, 122, 124, 124, 25, 123, 121 | **0.7304** |
| rps14 | 14, 32, 25, 49, 79, 9, 30, 39, 42, 36 | 14, 32, 25, 48, 78, 9, 30, 38, 41, 35 | **0.7132** |
| rps15 | 10, 86, 46, 31, 84, 83, 75, 16, 65, 74 | 0, 74, 34, 19, 72, 71, 63, 4, 53, 62 | **0.6015** |
| rps16 | 55, 17, 48, 35, 37, 46, 14, 33, 51, 13 | 54, 16, 47, 34, 36, 45, 14, 32, 50, 13 | **0.6548** |
| rps18 | 42, 138, 174, 9, 16, 28, 15, 183, 75, 182 | 35, 124, 157, 9, 16, 21, 15, 159, 61, 159 | **0.8346** |
| rps19 | 12, 16, 29, 3, 86, 23, 85, 18, 21, 64 | 12, 16, 29, 3, 86, 23, 85, 18, 21, 64 | **0.7695** |
| rpoA | 326, 14, 329, 6, 252, 180, 272, 234, 342, 155 | 317, 14, 320, 6, 246, 176, 263, 230, 333, 155 | **0.7416** |
| rpoB | 286, 273, 86, 266, 449, 594, 39, 995, 810, 722 | 284, 271, 86, 264, 447, 592, 39, 990, 805, 717 | **0.7495** |
| rpoC1 | 571, 579, 581, 348, 21, 79, 33, 549, 544, 651 | 566, 573, 575, 345, 21, 79, 33, 546, 541, 642 | **0.7951** |
| rpoC2 | 1402, 1290, 541, 1051, 371, 1343, 1050, 613, 1397, 401 | 1261, 1151, 538, 943, 371, 1202, 942, 607, 1256, 401 | **0.8242** |
| ycf3 | 34, 154, 44, 45, 175, 176, 20, 77, 57, 30 | 34, 148, 42, 42, 169, 170, 20, 74, 54, 30 | **0.558** |
| ycf4 | 103, 164, 119, 40, 44, 118, 101, 67, 37, 53 | 103, 164, 119, 40, 44, 118, 101, 67, 37, 53 | **0.6849** |

ap – positions numbered according to alignment position.

*Z. m* – Residue positions numbered according to positions in *Zea mays* protein sequence.

Acc – Accuracy after cross validation (using repeated random sub-sampling, n=500, 70/30 train/test split) is shown.

**
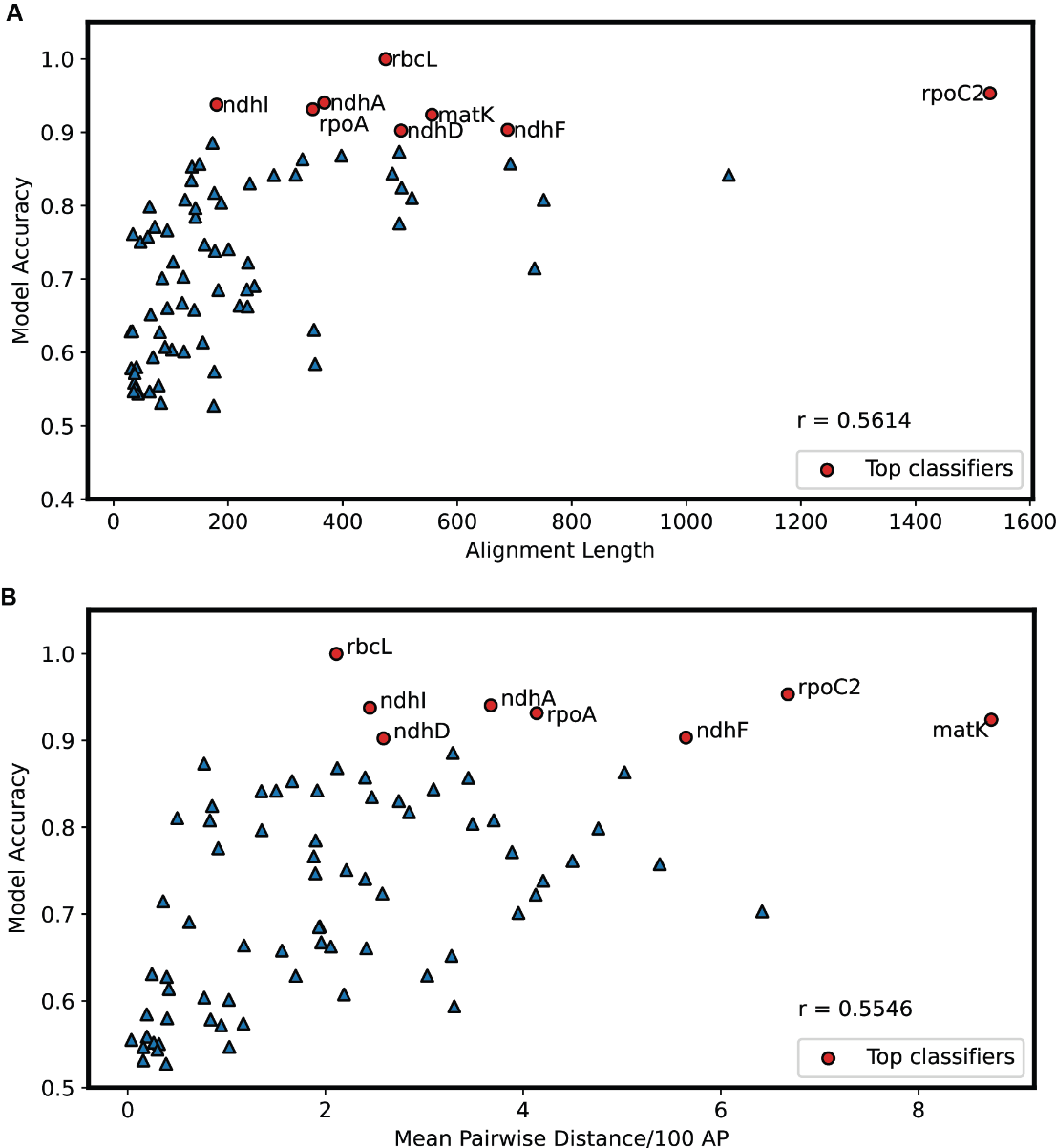
**

**Figure S1.** Optimal (v3) model accuracy correlation analyses. Average accuracy of the best performing model informed by each sequence, from iterations making use of <11 features, is plotted against corresponding (A) initial sequence alignment length and (B) mean pairwise distance per 100 alignment positions (MPD/100 AP) across C_3_ species of our dataset. Pearson’s correlation coefficient (r) is determined for both.


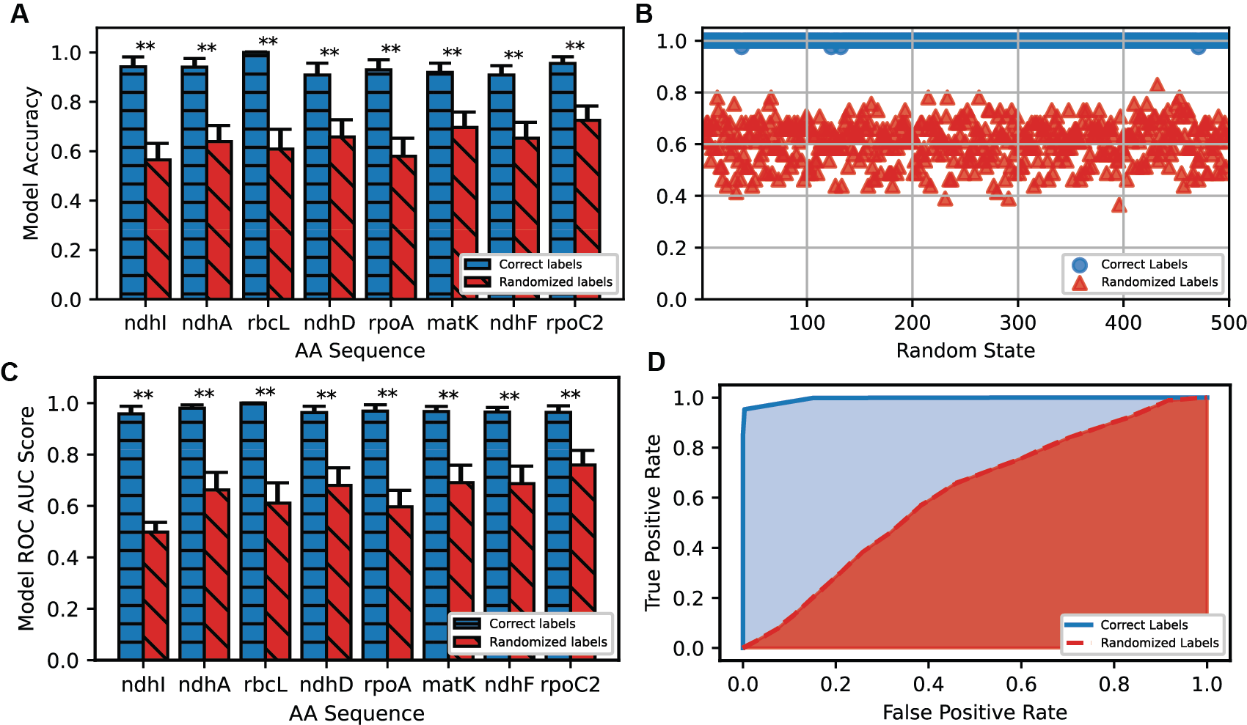


**Figure S2.** Summary and evaluation of optimal model performance. (A) Classification accuracies of optimal models after cross validation (true models) are compared here to classification accuracies of models trained with randomly assigned C_3_/C_4_ labels (random-label model). (B) Sample cross validation summary of optimal RbcL model. 500 different and random sub-samplings (70%) of the RbcL optimal features dataset are used to generate training sets for 500 classification models and their accuracies in classifying respective test sets (30%) are plotted (blue circles). This is compared to 500 models trained using optimized feature sets from predicting randomly assigned labels (red triangles). (C) Average ROC AUC scores, after cross validation, of true models and random-label models are compared. (D) Average ROC curves, after cross-validation, of RbcL true models (solid blue line) and random-label models (-- red line) are compared. Area under curves (AUC) are shaded to represent the respective average ROC AUC scores. ** Indicates significant difference in performance of true model and associated random-label model. Significance was determined using Student’s T-Tests (n=500, **p<0.001).


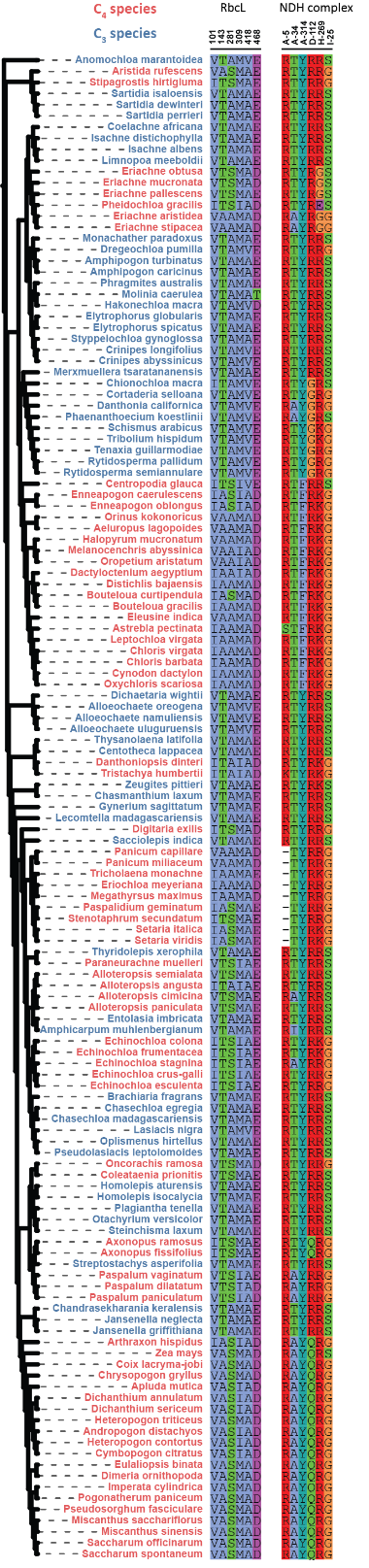


**Figure S3**. Mapping of residues from optimal feature positions (relative to *Zea mays*) to phylogenetic tree of 134 PACMAD species of our dataset (**Table S1**). Phylogeny was obtained using RAxML (GTR+Γ model) (Stamatakis 2014) from concatenated alignments of plastid marker genes (*rbcL*, *ndhF,* *matK, rpoC2, psaA, psaB, psaI, rpl32* and *ccsA*) and rooted to *Anomochloa marantoidea*, a grass species existing outside of the PACMAD clade. C_4_ species and C_3_ species are denoted by red and blue text, respectively. Residues from identified optimal feature positions (relative to *Zea mays*) of RbcL and the NDH complex are mapped to corresponding species. NDH complex position labels indicate Ndh subunit and position in subunit (subunit-position). – indicates a deletion at the specified position.

**
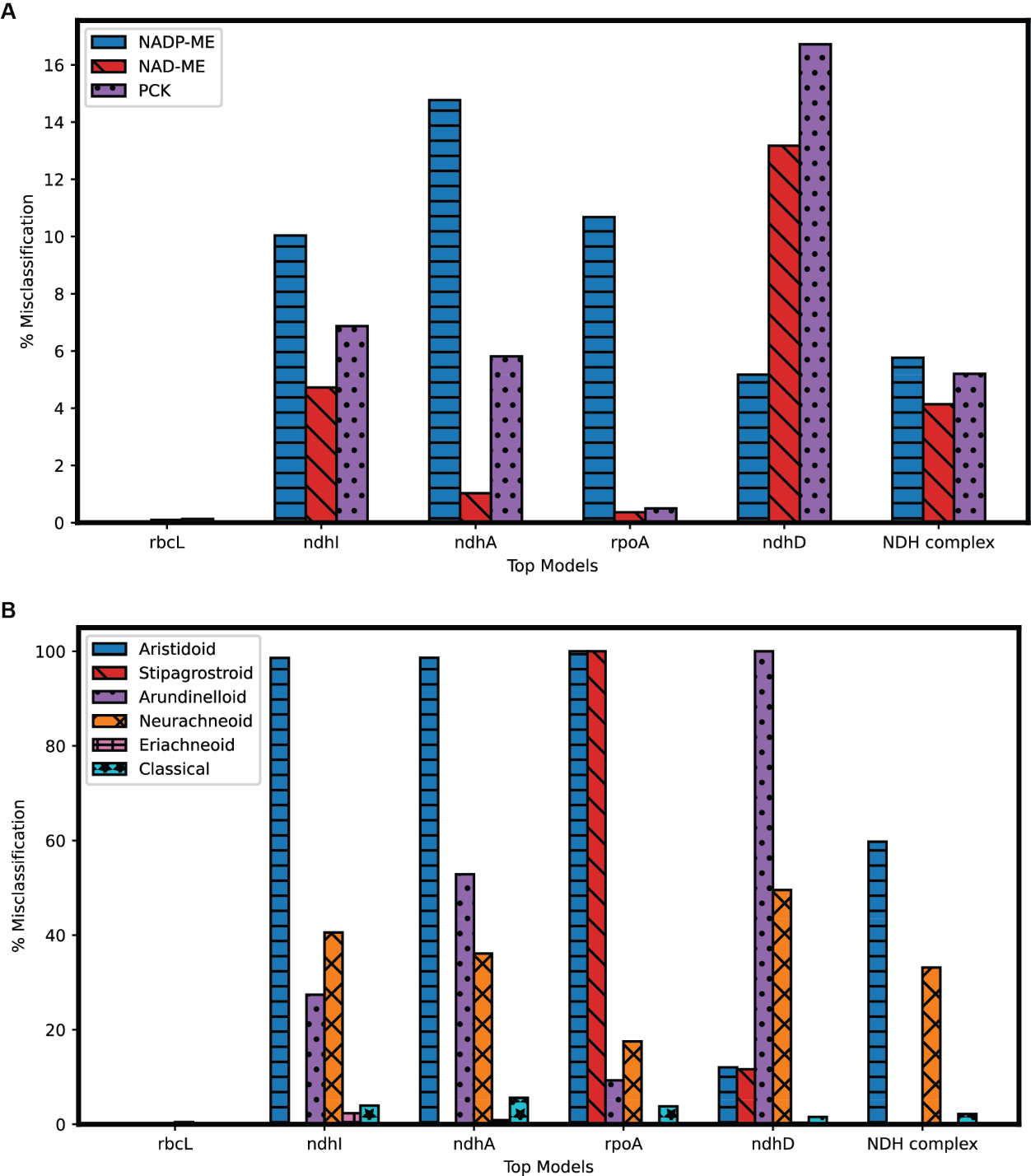
**

**Figure S4.** Misclassification rates of C_4_ species organized by associated (A) Biochemical type and (B) Kranz-type. Rates as a measure of the proportion of C_4_ species of a particular biochemical/kranz type that are misclassified as C_3_ species out of the total number of the particular biochemical/kranz type from across testing sets are shown.

**Table S3.** List of Eudicot species in assembled Eudicot dataset used in this study. C_3_/C_4_ status, Kranz-type, and Biochemical type are determined as referenced in Sage et al. (2011) and from Muhaidat et al. (2007), Edwards and Vosnesenskya (2011), and Rudov et al. (2020). Typing from latest study is used for conflicting reports.

| **Order** | **Family** | ***Species*** | **C3/C4** | **Kranz type** | **Biochemical type** |
| --- | --- | --- | --- | --- | --- |
| Asterales | Asteraceae | *Flaveria bidentis* | C4 | Atriplicoid | NADP-ME |
|  |  | *Helianthus annuus* | C3 |  |  |
| Brassicales | Cleomeaceae | *Cleome gynandra (Gynandropsis gynandra)* | C4 | Atriplicoid | NAD-ME |
|  |  | *Cleome pallida* | C3 |  |  |
| Caryophyllales | Aizoaceae | *Aptenia cordifolia* | C3 |  |  |
|  |  | *Sesuvium portulacastrum* | C3 |  |  |
|  | Amaranthaceae | *Achyranthes longifolia* | C3 |  |  |
|  |  | *Achyranthes aspera* | C3 |  |  |
|  |  | *Amaranthus caudatus* | C4 | Atriplicoid | NAD-ME |
|  |  | *Amaranthus hybridus* | C4 | Atriplicoid | NAD-ME |
|  |  | *Amaranthus hypochondriacus* | C4 | Atriplicoid | NAD-ME |
|  |  | *Amaranthus tricolor* | C4 | Atriplicoid | NAD-ME |
|  |  | *Celosia cristata* | C3 |  |  |
|  |  | *Cyathula capitata* | C3 |  |  |
|  |  | *Deeringia amaranthoides* | C3 |  |  |
|  |  | *Froelichia latifolia* | C4 | Atriplicoid | NADP-ME |
|  |  | *Ptilotus polystachyus* | C3 |  |  |
|  | Chenopodiaceae | *Atriplex centralasiatica* | C4 | Atriplicoid | NAD-ME |
|  |  | *Atriplex gmelinii* | C3 |  |  |
|  |  | *Chenopodium quinoa* | C3 |  |  |
|  |  | *Spinacia oleracea* | C3 |  |  |
|  | Chenopodiaceae/  Salsoloideae | *Caroxylon passerinum* | C4 | Salsoloid | NAD-ME |
|  |  | *Haloxylon ammodendron* | C4 | Salsoloid | NAD-ME |
|  | Gisekiaceae | *Gisekia pharnaceoides* | C4 | Atriplicoid | NAD-ME |
|  | Nyctaginaceae | *Boerhavia diffusa* | C4 | Atriplicoid | NADP-ME |
|  |  | *Pisoniella arborescens* | C3 |  |  |
|  | Polygonaceae | *Atraphaxis bracteata* | C3 |  |  |
|  |  | *Calligonum mongolicum* | C4 | Salsoloid | NAD-ME |
|  | Portulaceae | *Portulaca grandiflora* | C4 | Portulacenoid | NAD-ME |
|  |  | *Portulaca oleracea* | C4 | Portulacenoid | NAD-ME |
| Lamiales | Acanthaceae | *Blepharis cilliaris* | C4 | Atriplicoid | NAD-ME |
|  |  | *Justicia adhatoda* | C3 |  |  |
| Malpighiales | Euphorbiaceae | *Euphorbia hirta* | C4 | Atriplicoid | NADP-ME |
|  |  | *Euphorbia maculata* | C4 | Atriplicoid | NADP-ME |
| Zygophyllales | Zygophyllaceae | *Larrea tridentata* | C3 |  |  |
|  |  | *Tribulus terrestris* | C4 | Atriplicoid | NADP-ME |

**Table S4.** Summary of model performances after feature selection - using recursive feature elimination with cross validation - from Eudicot-derived sequence data.

| **AA seq** | **Optimal Features (ap)** | **Residue Positions (*Zm*)** | **Acc** |
| --- | --- | --- | --- |
| RbcL | 29, 97, 103, 105, 236, 259, 289, 317, 336 | 21, 89, 95, 97, **228**, 251, **281**, **309**, **328** | **0.8356** |
| NdhI | 85, 89, 95, 149, 169, 170 | **85**, **89**, 95, 149, 168, 169 | **0.6547** |
| NdhA | 5, 14, 123, 139, 183, 318 | **5**, 12, 120, 136, 180, 311 | **0.8558** |
| RpoA | 76, 244, 254, 309, 329, 343, 347 | 68, **237**, **246**, 301, **321**, **332**, 336 | **0.8687** |
| NdhD | 45, 102, 216, 323, 467 | 15, 72, 186, 293, 437 | **0.8421** |

ap – positions numbered according to alignment position.

*Z. m* – Residue positions numbered according to positions in *Zea mays* protein sequence.

Acc – Accuracy after cross validation (using repeated random sub-sampling, n=500, 70/30 train/test split) is shown.

Highlighted residue positions indicate matches (within 1 residue position) to identified strongest/optimal features from corresponding v2 and v3 models bult on Poaceae sequences.


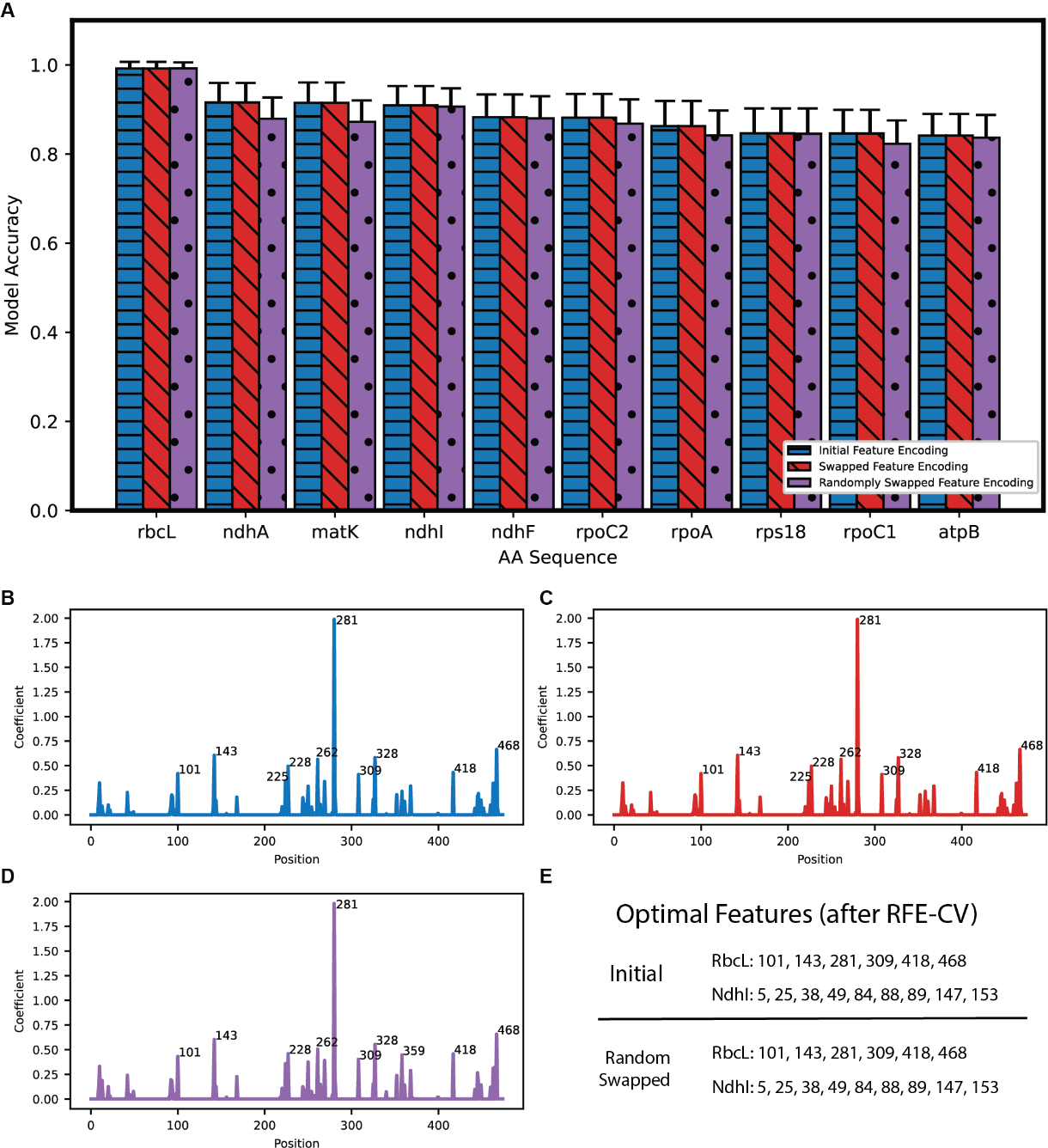


**Figure S5.** Effects of feature engineering method on classification accuracy and the identification of informative features. (A) Average classification accuracies of v1 models after cross validation are compared here to assess the effect of swapping amino acid encodings at each alignment position. Original models trained on initial feature encodings are compared to models trained on features with oppositely swapped amino acid encodings (see methods for details), and to models trained on features with randomly swapped amino acid encodings. (B), (C), (D) are feature importance profiles as parsed from logistic regression classifiers trained on RbcL alignment data using the initial feature encodings, swapped feature encodings, and randomly swapped feature encodings respectively. The 10 strongest features corresponding to the 10 highest peaks are labelled in each plot. (E) Comparison of optimal features identified from RbcL and NdhI models after recursive feature elimination with cross validation (RFE-CV) and trained on initial feature encodings or randomly swapped feature encodings.

**Table S5.** Summary of feature identification and model performance using recursive feature elimination with cross validation (90% of dataset) followed by validation (10% of dataset)

| **AA seq** | **Optimal Features (ap)** | **Residue Positions (*Zm*)** | **Train/Test Accuracy** | **Validation Accuracy** |
| --- | --- | --- | --- | --- |
| RbcL | 101, 143, 281, 309, 418, 468 | 101, 143, 281, 309, 418, 468 | **0.999** | **1.0** |
| NdhI | 5, 25, 38, 49, 84, 88, 89, 147, 153 | 5, 25, 38, 49, 84, 88, 89, 147, 153 | **0.926** | **1.0** |
| NdhA | 5, 11, 29, 36, 98, 110, 294, 302, 319, 320 | 5, 11, 27, 34, 96, 108, 289, 297, 314, 315 | **0.921** | **1.0** |
| RpoA | 14, 146, 163, 180, 229, 236, 243, 326, 329, 336 | 14, 146, 161, 176, 225, 232, 237, 317, 320, 327 | **0.9329** | **0.786** |
| NdhD | 64, 76, 109, 201, 266, 318, 376, 401, 442, 451 | 62, 74, 107, 199, 264, 316, 374, 440, 449 | **0.8433** | **0.857** |

ap – positions numbered according to alignment position.

*Z. m* – Residue positions numbered according to positions in *Zea mays* protein sequence.

Train/Test Accuracy – Accuracy after cross validation (using repeated random sub-sampling, n=500, 70/30 train/test split – on 90% of dataset) is shown.

Validation Accuracy – Accuracy of trained model using identified optimal features on validation set (10% of dataset) is shown.

Highlighted residue positions indicate matches to identified optimal features after recursive feature elimination with nested cross validation on 100% of our PACMAD dataset
